# Developing a multi-epitope vaccine candidate to combat porcine epidemic diarrhea virus and porcine deltacoronavirus co-infection by employing an immunoinformatics approach

**DOI:** 10.3389/fmicb.2023.1295678

**Published:** 2023-11-21

**Authors:** Wei Hou, Heqiong Wu, Wenting Wang, Ruolan Wang, Wang Han, Sibei Wang, Bin Wang, Haidong Wang

**Affiliations:** ^1^College of Veterinary Medicine, Shanxi Agricultural University, Jinzhong, China; ^2^Single Molecule Nanometry Laboratory (Sinmolab), Nanjing Agricultural University, Nanjing, China

**Keywords:** PEDV, PDCoV, vaccine, multi-epitope, immunoinformatics

## Abstract

Coinfection of porcine epidemic diarrhea virus (PEDV) and porcine deltacoronavirus (PDCoV) is common in pig farms, but there is currently no effective vaccine to prevent this co-infection. In this study, we used immunoinformatics tools to design a multi-epitope vaccine against PEDV and PDCoV co-infection. The epitopes were screened through a filtering pipeline comprised of antigenic, immunogenic, toxic, and allergenic properties. A new multi-epitope vaccine named *rPPMEV*, comprising cytotoxic T lymphocyte-, helper T lymphocyte-, and B cell epitopes, was constructed. To enhance immunogenicity, the TLR2 agonist Pam2Cys and the TLR4 agonist RS09 were added to *rPPMEV*. Molecular docking and dynamics simulation were performed to reveal the stable interactions between *rPPMEV* and TLR2 as well as TLR4. Additionally, the immune stimulation prediction indicated that *rPPMEV* could stimulate T and B lymphocytes to induce a robust immune response. Finally, to ensure the expression of the vaccine protein, the sequence of *rPPMEV* was optimized and further performed *in silico* cloning. These studies suggest that *rPPMEV* has the potential to be a vaccine candidate against PEDV and PDCoV co-infection as well as a new strategy for interrupting the spread of both viruses.

## 1 Introduction

Currently, the alphacoronavirus porcine epidemic diarrhea virus (PEDV) and deltacoronavirus porcine delta coronavirus (PDCoV) are two main swine enteric coronaviruses (Koonpaew et al., 2019; Hou et al., 2023): the former can infect swine of all ages and cause watery diarrhea, vomiting, and dehydration, and the latter causes acute diarrhea, vomiting, and dehydration in neonatal piglets (Tang et al., 2021; Hou et al., 2023). Especially, the co-infection of the two viruses, which both continue to emerge and reemerge worldwide, causing more severe mortality and economic losses.

To rapidly and efficiently prevent and control PEDV and PDCoV co-infection, the vaccine is a valuable means (Trovato et al., 2020). As a vaccine development route, the traditional methods are time-consuming and labor-intensive (Nabel, 2002), and such vaccines often contain large proteins or the entire organism, resulting in an unnecessary antigenic load and increasing the likelihood of eliciting an allergic reaction (Chauhan et al., 2019). These problems can be solved by using peptide-based vaccines, which are made up of brief immunogenic peptide fragments that can elicit highly targeted immune responses, thereby reducing the likelihood of an allergic reaction. In peptide-based vaccine development, effective screening and immunogen design are major challenges since short peptides typically have weak immunogenic effects due to their small molecular weights (Sun et al., 2022). According to reported works, the coronavirus S protein plays an important role in viral entry and virus-host interaction, and it is the primary target for stimulating the host cell immune response and inducing neutralizing antibodies (Sun et al., 2008; Li et al., 2021). Furthermore, it was also reported that the S proteins of PEDV and PDCoV have good immunity and the potential for vaccine development (Wang et al., 2016; Zhai et al., 2023). Therefore, the S protein is the preferred region for immunogen screening and the design of the PEDV and PDCoV vaccine. For immunogen screening, the typical epitope screening is to insert a peptide with the target epitope into the plasmid and verify the immune effect of the epitope through large experiments (De Groot et al., 2001). Immunoinformatics approaches, which can eliminate the need for time-consuming and expensive manipulation as well as complex procedures, have emerged as a crucial tool for epitope localization and are playing an increasingly important role in epitope discovery as well as in successful vaccine design (Khan et al., 2018; Dong et al., 2020). Khan et al. (2021) used immunoinformatics methods to design a universal multi-epitope vaccine against SARS-CoV-2. Rowaiye et al. (2023) developed a multiepitope vaccine candidate to curb the outbreaks of African swine fever virus using the immunoinformatics. Therefore, these immunoinformatics approaches can be employed for vaccine design for the PEDV and PDCoV co-infection.

In this study, we employed immunoinformatic approaches to predict and design a safe and effective multi-epitope candidate vaccine derived from the S protein for prevalent PEDV and PDCoV variants. The designed vaccine named *rPPMEV* comprises a range of predicted epitopes, can interact with TLRs, and has the potential to stimulate T and B lymphocytes to induce a strong immunological response. The findings of this study provide a new vaccine candidate for the prevention of PEDV and PDCoV co-infection.

## 2 Materials and methods

**Figure 1:**
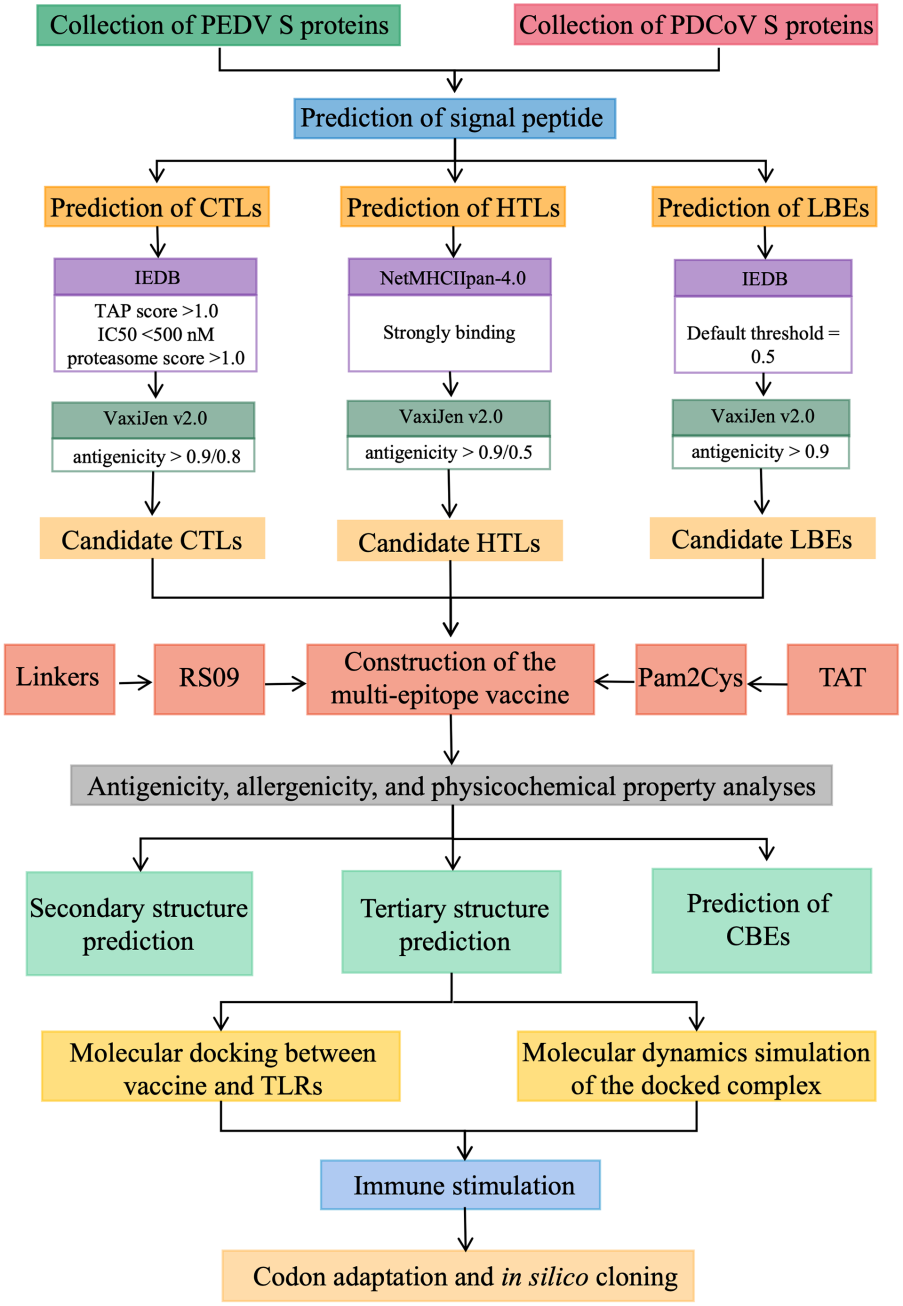
The prediction procedures of the multi-epitope candidate vaccine for PEDV and PDCoV co-infection.

To predict and design a safe and effective multi-epitope candidate vaccine for PEDV and PDCoV co-infection, procedures listed in Figure 1 were implemented. In this section, these procedures are briefly mentioned below.

### 2.1 Identification of target antigens

PEDV strains CH/HLJJS/2022, CH/HBXT/2018, and SXSL were selected since they have been highly pathogenic in recent years (Zhang et al., 2019; Liu et al., 2022; Yao et al., 2023). The spike (S) glycoproteins UUT43943.1, AZL49329.1, and UWU45211.1 of these three PEDV strains were selected as candidate antigens for epitope prediction due to their significant usefulness in PEDV vaccines (Zhang et al., 2019, Liu et al., 2022, Yao et al., 2023). In addition, PDCoV strains CH-HLJ-20, HNZK-02, Swine/CHN/SC/2018/1, CHN/Sichuan/2019-MK993519.1, and CHN-TS1-2019-MT663769.1 were selected for vaccine development as these have been prevalent in recent years (Kong et al., 2022). The S glycoproteins QZA57171.1, AXP32216.1, QCO76963.1, QGZ00525.1, and QZX45753.1 of these five PDCoV strains were selected as preparatory antigens because the S protein has good immunity for PDCoV vaccine design (Zhai et al., 2023). The sequences of all S proteins were obtained from the National Center for Biotechnology Information (NCBI) database.^1^

### 2.2 Prediction of signal peptide

To determine whether the signal peptide is present in the candidate antigen proteins, the signal peptide of the S protein was predicted using SignalP-5.0 server^2^ (Almagro Armenteros et al., 2019). The following T and B cell epitope predictions all need to remove the signal peptides.

### 2.3 Prediction of cytotoxic T lymphocyte epitopes

The Immune Epitope Database (IEDB) server^3^ was used to predict cytotoxic T lymphocyte epitopes (CTLs) (Fleri et al., 2017). The epitope length of 9 residues was used to predict the epitope through 45 common swine leukocyte antigen (SLA) class I molecules by running the “IEDB-recommended” method. Then, the epitopes were screened using a TAP score >1.0, an IC50 <500 nM, and a proteasome score >1.0. The dominant epitopes, which simultaneously appeared in at least three SLA-I alleles in each viral strain and had high antigenicity (> 0.9 for PEDV, > 0.8 for PDCoV), were further predicted by using VaxiJen v2.0.^4^ Finally, the common dominant epitopes of the PEDV and PDCoV strains were used to construct the final vaccine.

### 2.4 Prediction of helper T lymphocyte epitopes

The online server NetMHCIIpan 4.0^5^ was used to predict helper T lymphocyte epitopes (HTLs). A length of 15 amino acid residues was used for epitope prediction through 27 high-frequency human MHC II (HLA-II) alleles (Ros-Lucas et al., 2020). The threshold for strongly binding peptides was set to its default value. The dominant epitopes, which simultaneously appeared in at least three HLA-II alleles in each viral strain and had high antigenicity (> 0.9 for PEDV, > 0.5 for PDCoV), were further predicted by using VaxiJen v2.0. Finally, the common dominant epitopes of the PEDV and PDCoV strains were used to construct the final vaccine.

### 2.5 Prediction of linear B cell epitopes

For the prediction of linear B cell epitopes (LBEs), the IEDB server with the method of Bepipred Linear Epitope Prediction 2.0 at the default threshold of 0.5 was used. Then, the predicted epitopes were screened using VaxiJen v2.0. Finally, the common epitopes with high antigenicity (>0.9) of the PEDV or PDCoV strains were used for vaccine construction.

### 2.6 Construction of the multi-epitope vaccine

The final subunit vaccine was constructed by sequentially combining the generated peptide sequences with appropriate linkers. To improve the antigenicity and immunogenicity of the vaccine, the toll-like receptor 4 (TLR4) agonist RS09 and the TLR2 agonist dipalmitoyl-S-*glycero*-cysteine (Pam2Cys) were added to the N-terminal and C-terminal via the EAAAK linker, respectively (Jackson et al., 2004; Meza et al., 2017; Albutti, 2021). The CTLs, HTLs, and B cell epitopes were joined by AAY, GPGPG, and KK, respectively. In addition, the TAT sequence (11 aa) was added to its carboxyl terminus to enhance the intracellular delivery of the vaccine (Frankel and Pabo, 1988).

### 2.7 Antigenicity, allergenicity, and physicochemical property analyses of the multi-epitope vaccine

The antigenicity and allergenicity of the multi-epitope vaccine were analyzed using the online software VaxiJen v2.0 and AllerTop v. 2.0,^6^ respectively. The physicochemical characteristics of the multi-epitope vaccine, such as its molecular weight, atomic composition, theoretical isoelectric point (PI), half-life, stability, hydropathicity, and other properties, were predicted using the online program Protparam.^7^

### 2.8 Prediction of the secondary and tertiary structures of the multi-epitope vaccine

The secondary structure of the multi-epitope vaccine was predicted using SOPMA online analysis software^8^ (Deléage, 2017). The initial tertiary structure was predicted by Robetta server^9^ (Baek et al., 2021). After primary 3D modeling, the initial tertiary structure was further optimized by GalaxyRefine server^10^ (Yu et al., 2022). Later, the refined structure was validated using two online tools: SWISS-MODEL workspace^11^ and ProSA-web.^12^ The SWISS-MODEL workspace was used to evaluate the quality of protein by analyzing the Ramachandran plot (Waterhouse et al., 2018). The ProSA-web was used for protein validation by generating a *z*-score (Wiederstein and Sippl, 2007).

### 2.9 Prediction of conformational B cell epitopes

The conformational B cell epitopes (CBEs) of the multi-epitope vaccine were predicted using the online software IEDB ElliPro tool^13^ with the default parameters of a minimum score of 0.5 and a maximum distance of 6 angstrom (Ponomarenko et al., 2008), and visualized with PyMol.

### 2.10 Molecular docking between the multi-epitope vaccine and TLRs

The molecular docking between the vaccine construct and the TLRs was performed using ClusPro server^14^ (Kozakov et al., 2017). The receptors were TLR2 (PDB ID: 6NIG) and TLR4 (PDB ID: 4G8A), and the ligand was the multi-epitope vaccine. The PDB file of the docking results was loaded into Ligplot and PyMol to analyze the interaction interface residues.

### 2.11 Molecular dynamics simulation of the docked complex

To understand any state changes in a given biological environment, the molecular dynamics (MD) simulation was applied to the TLR2-Vaccine and TLR4-Vaccine complexes using GROMACS (GROningen MAchine for Chemical Simulations) (He et al., 2021). First, in all MD simulations, the protein-ligand complex architecture was generated using the AMBER99 force field. The protein was then solvated in a cubic box of TIP3P waters (Grifoni et al., 2020), with a minimum distance of 1.0 nm (TLR2-Vaccine, TLR4-Vaccine) between the protein and box edge (Ismail et al., 2020). The charged protein complex was neutralized by the addition of ions using a genion tool (Shukla et al., 2018). Additionally, the solvated electroneutral system was relaxed through energy minimization in order to avoid steric conflicts and inappropriate geometry. Then, 100 ps of NVT [substance (N), volume (V), and temperature (T)] equilibration and 100 ps of NPT [substance (N), pressure (P), and temperature (T)] equilibration were used to acclimate the system without restrictions. After proper minimizations and equilibrations, a productive MD run of 20 ns was performed for all the complex systems, and the parameters, root mean square deviation (RMSD) and root mean square fluctuation (RMSF), which define the stability of the docked complex on simulation, were computed.

### 2.12 Immune stimulation

To detect the immune response of the multi-epitope vaccine to the host, the C-ImmSim server^15^ was used for the immune simulation (Rapin et al., 2010). The time steps were set at 1, 84, and 168 (one time step corresponds to 8 h). The number of simulation steps was set at 1,050 (Bhatnager et al., 2021). The other parameters were used as the default simulation parameters.

### 2.13 Codon adaptation and *in silico* cloning

To achieve superior expression of recombinant protein, the codon adaptation of the multi-epitope vaccine was performed by the online tool Java Codon Adaptation Tool (JCat)^16^ (Grote et al., 2005). *Escherichia coli* (Strain K12) was chosen to express the vaccine protein. The indicators, codon adaptation index (CAI) the ideal value is (1) and percentage GC content (the ideal range is 30%–70%), were analyzed (Puigbo et al., 2007). For *in silico* cloning of the vaccine construct, pET28a (+) was selected as the vector. The codon-optimized sequence of the vaccine was cloned into the vector through the *XhoI* and *BamHI* restriction sites by SnapGene tool.^17^

## 3 Results and discussion

### 3.1 The acquisition of vaccine-candidate antigens

**Figure 2:**
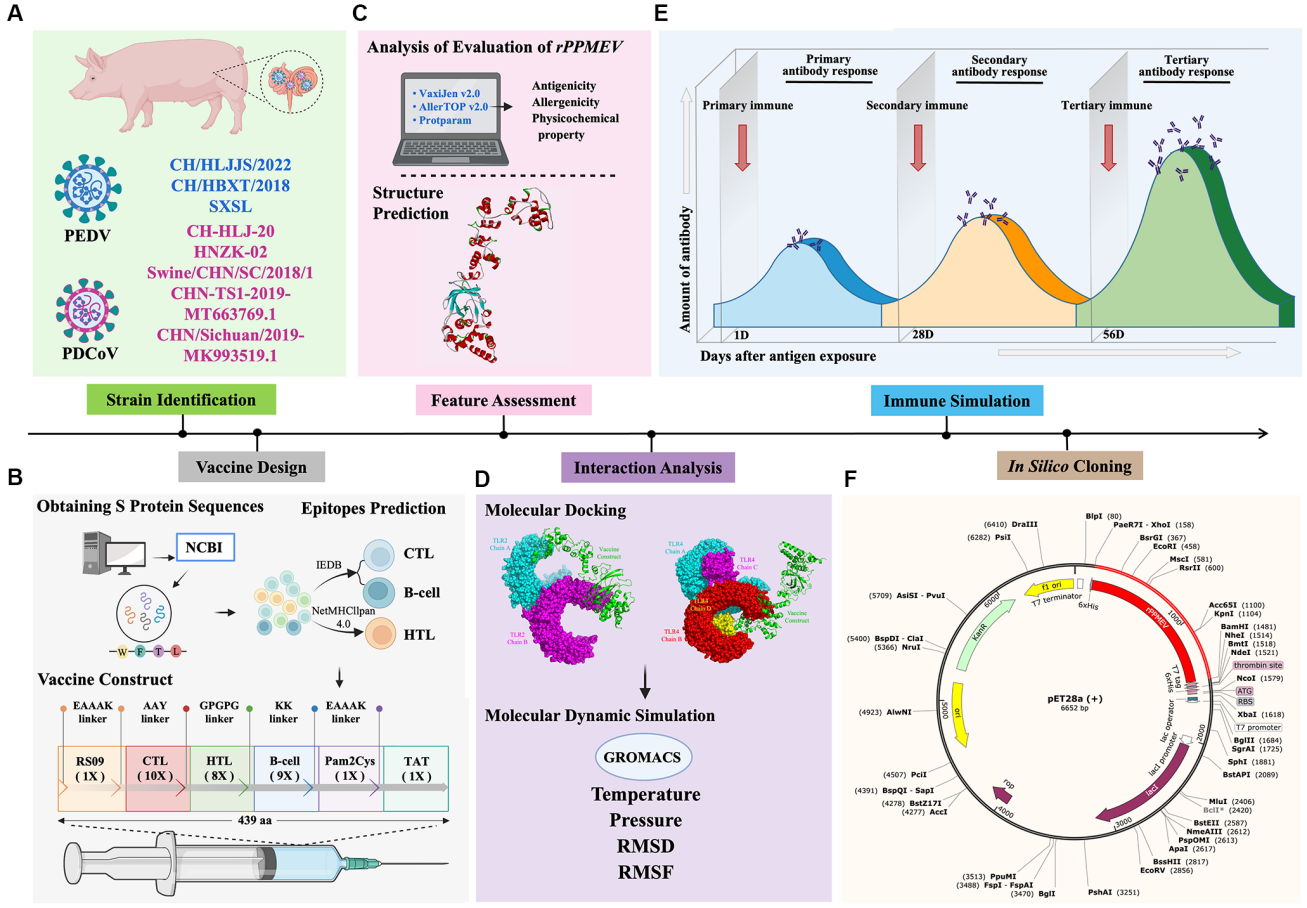
Flow chart of the multi-epitope selection and the final rPPMEV construction. The rPPMEV was designed in six steps with different colors, including PEDV and PDCoV strain identification **(A)**, vaccine design **(B)**, *rPPMEV* feature assessment **(C)**, the interaction analysis of *rPPMEV* with TLR2 and TLR4 immune receptors **(D)**, *rPPMEV* immunological characteristics analysis **(E)**, and *in silico* cloning **(F)**.

Nowadays, the co-infection of PEDV and PDCoV, which both continue to emerge and reemerge worldwide, causes massive economic losses to the swine industry globally (Jiao et al., 2021). To rapidly and efficiently prevent and control virus infection, the development of vaccines has become imperative. In this study, we used immunoinformatics to discover and design a multivalent epitope vaccine to combat PEDV and PDCoV. The schematic procedure of the multi-epitope selection and the final vaccine construction is shown in Figure 2. The development of a new vaccine derived from a highly virulent virus provides cross-protection against low-virulence virus infection (Yao et al., 2023), and a vaccine developed from the strains responsible for the current outbreak will be successful in preventing viral infection (Rock, 2017; Borca et al., 2020; Moise et al., 2020). Thus, the three highly pathogenic PEDV strains and five prevalent PDCoV strains were selected for vaccine development. Furthermore, because the S proteins of PEDV and PDCoV strains have strong immunogenicity and the potential to generate vaccines (Wang et al., 2016; Zhai et al., 2023), the S proteins were selected as the preparatory antigens for immunogen screening and the design of a new PEDV and PDCoV vaccine. The GeneBank accession numbers of the three PEDV strains and five PDCoV strains, as well as the antigenicity of all S proteins, are shown in Supplementary Table S1, and the amino acid sequence of all S proteins is shown in Supplementary Data.

**Figure 3:**
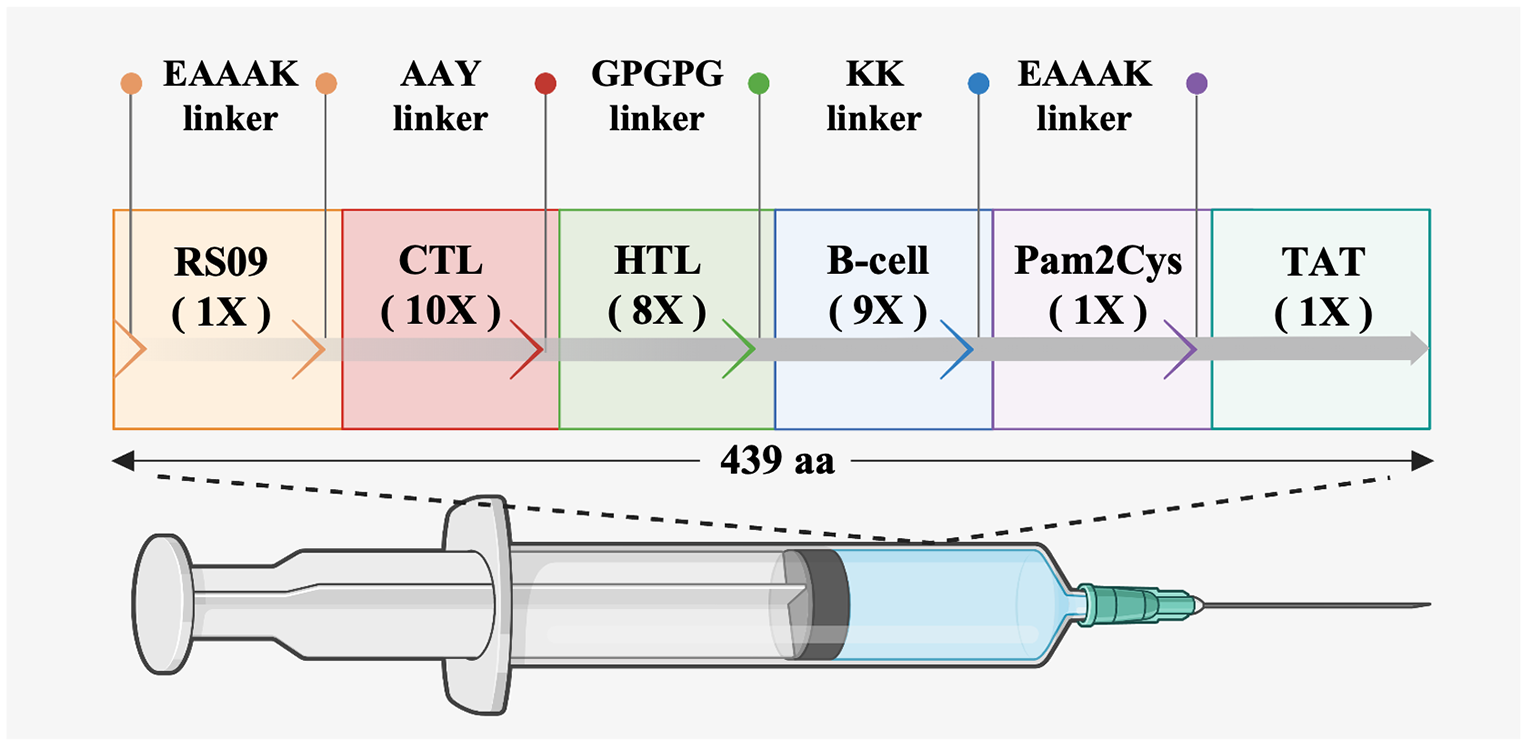
The design and construction of *rPPMEV*. The *rPPMEV* contains 439 amino acids, and the components needed in *rPPMEV* construction are represented in different colors.

To prepare an epitope vaccine, obtaining the epitopes of the relative antigen is the key point (Li et al., 2013). Firstly, to determine whether these S proteins contain signal peptide regions, the signal peptide was examined before the epitope prediction. The findings reveal that the signal peptide sequence of PEDV is 1–18 (MKSLTYFWLLLPVLSTLS), while the signal peptide region of PDCoV is 1–19 (MQRALLIMTLLCLVRAKFA) (Supplementary Table S1). Then, to avoid specifying or inhibiting protein localization, the signal peptide sequences were removed from the epitope prediction of all S proteins (Mahmud et al., 2021). Secondly, it was reported that cytotoxic T cells are important for specific antigen recognition and the helper T cells are an essential component of adaptive immunity, which function in activating B cells, macrophages, and even cytotoxic T cells (Dimitrov et al., 2013; Gupta et al., 2013), the two types of epitopes, CTLs and HTLs, of T cell epitopes were predicted in this study. Furthermore, the B cell epitope was also screened for the vaccine construct since it could trigger the production of antigen-specific immunoglobulins, which are crucial components of adaptive immunity (Sarkar et al., 2022). After epitope prediction, the inclusion criteria for immunodominant epitopes are as follows: (1) the common dominant CTLs simultaneous appearance in at least three SLA-I alleles of each PEDV strain or PDCoV strain and with a high antigenicity score; (2) the common dominant HTLs simultaneous appearance in at least three HLA-II alleles in each PEDV strain or PDCoV strain and with a high antigenicity score; (3) the common dominant LBEs simultaneous appearance in each PEDV or PDCoV strain and with high antigenicity. Finally, 10 CTLs, 8 HTLs, and 9 LBEs were chosen for constructing the new multi-epitope vaccine *rPPMEV* (Supplementary Table S2). Then, according to the reports, TLRs are constitutively expressed in innate immune cells and play a vital role in viral recognition, leading to antiviral signaling cascades. Specifically, cell membrane receptors TLR2 and TLR4 play an important role in recognizing envelope glycoproteins (Kurt-Jones et al., 2000; Boehme and Compton, 2004; Takeda and Akira, 2004; Barton, 2007; Temeeyasen et al., 2018). To significantly improve the immunogenicity and antigenicity of *rPPMEV*, the TLR2 agonist Pam2Cys (FNNFTVSFWLRVPKVSASHLE) and TLR4 agonist RS-09 (APPHALS), which can trigger activation of TLR2 and TLR4 signaling, respectively (Jiang et al., 2023), were incorporated into the vaccine design. Additionally, to conjugate and enhance expression function and prevent the production of neo-epitopes, linker selection is a significant concern in the development of multi-epitope vaccines (Chen et al., 2013). The EAAAK linker, which can effectively separate and decrease interaction between vaccines as well as increase the thermal stability of the chimeric protein (Saadi et al., 2017), was used to join the adjuvants RS-09 and Pam2Cys. The AAY, GPGPG, and KK linkers were utilized to join the CTLs, HTLs, and B cell epitopes, respectively. The TAT sequence was attached to the C-terminal of the vaccine construct to enhance vaccine intercellular delivery. Finally, the new multivalent vaccine, *rPPMEV*, which was designed using immunoinformatics techniques, has a length of 439 amino acids, as illustrated in Figure 3.

### 3.2 Allergenicity, antigenicity, and physicochemical property analyses of *rPPMEV*

To evaluate the safety of *rPPMEV*, an allergenicity analysis was carried out using the AllerTop v. 2.0 server. The results show that *rPPMEV* and its closest protein (UniProtKB accession number O14514) are non-allergic. Moreover, the antigenicity analysis of VaxiJen v2.0 reveals that *rPPMEV* exhibits strong antigenicity with a score of 0.7241, which is above the threshold of 0.4. These results demonstrate that *rPPMEV* is safe for administration to swine. Additionally, the physicochemical properties of *rPPMEV* were also analyzed, as the physical properties of proteins significantly affect their immune function (Ikai, 1980). The finding shows that *rPPMEV* has 439 amino acids, 6,617 total atoms, the formula C_2154_H_3274_N_554_O_627_S_8_, and a molecular weight of 47 KD, which can be easily purified since the molecular weight of the protein is less than 110 KD (Barh et al., 2013). The theoretical pI of *rPPMEV* is 9.39, and it includes 28 negatively charged residues and 46 positively charged residues. The instability index of *rPPMEV* was calculated to be 33.81 (a value below the threshold value of 40 means that the protein is stable), indicating that *rPPMEV* should be stable upon expression in host systems. Furthermore, the aliphatic index of *rPPMEV* is 68.54, and the Grand average of hydropathicity (GRAVY) of *rPPMEV* is-0.249 (the range of GRAVY is −2 to 2, a negative value means that protein is hydrophilic) (Sha et al., 2020), showing that *rPPMEV* is hydrophilic.

### 3.3 The prediction of *rPPMEV* secondary and tertiary structure

**Figure 4:**
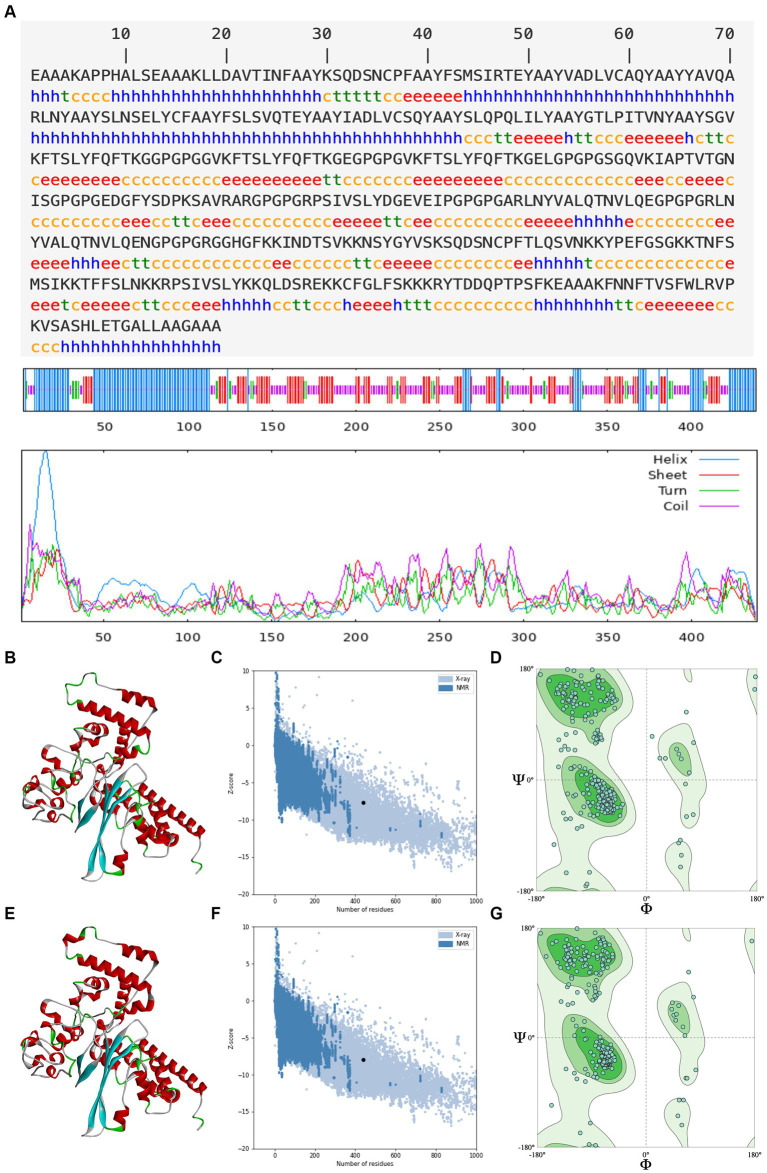
The prediction of *rPPMEV* secondary and tertiary structure. **(A)** The prediction of *rPPMEV* secondary structure. The blue “h” represents the alpha helix, the red “e” represents the extended strand, the green “t” represents the beta turn, and the yellow “c” represents the random coil. **(B)** The prediction of *rPPMEV* initial tertiary structure. The alpha helix, extended strand, beta turn, and random coil are marked in the 3D model with the colors of red, cyan, green, and gray, respectively. **(C)** The *z*-score of the *rPPMEV* initial tertiary structure. **(D)** The Ramachandran plots of the *rPPMEV* initial tertiary structure. **(E)** The prediction of the *rPPMEV* final tertiary structure. In the 3D model, the “red,” “cyan,” “green,” and “gray” parts represent alpha helix, extended strand, beta turn, and random coil, respectively. **(F)** The *z*-score of the *rPPMEV* final tertiary structure. **(G)** The Ramachandran plots of the *rPPMEV* final tertiary structure.

An ideal peptide-based vaccination designed using immunoinformatics techniques should trigger a strong immunological response without having any negative side effects (Tahir ul Qamar et al., 2020; Shantier et al., 2022). The secondary structure determines the stability of protein structure, which is essential for antigen proteolysis, presentation, and activation of T and B cells (Scheiblhofer et al., 2017), and the tertiary structure determines the molecular recognition by the TCR (Greenbaum et al., 2007). As a result of secondary prediction, there is 31.89% alpha helix, 25.06% extended strand, 7.52% beta turn, and 35.54% random coil in *rPPMEV*, as shown in Figure 4A. Among these regions, the naturally unfolding protein regions and alpha-helical coiled coils, as basic types of “structural antigens,” can induce antibody recognition after infection (Corradin et al., 2007). Subsequently, the tertiary structure of the vaccine was predicted using the Robetta server. There are five models outputted in the result. The *z*-score was calculated on all models through ProSA-web. The *z*-scores of models 1–5 are −7.43, −7.64, −6.92, −6.19, and −6.59, respectively, as shown in Supplementary Figure S1. Model 2 was selected as the initial model of *rPPMEV* (Figure 4B) since it has the highest quality with the lowest *z*-score (Figure 4C). The Ramachandran plot shows that Model 2 has 91.53% favored region, 1.60% outlier region, and 0.00% rotamer region (Figure 4D). To improve the structure quality and protein stability, the initial model was refined by the GalaxyRefine server. As a result, five optimized 3D models are presented. The *z*-scores of these optimized models 1–5 are −7.84, −7.69, −7.62, −7.93, and −7.66, respectively (Supplementary Figure S2). Similar to the initial model selection, the optimized Model 4 was adopted as the final tertiary structure of *rPPMEV* (Figure 4E), which has the lowest *z*-score (Figure 4F) and performs at 93.59%, 1.14%, and 1.16% in the favored, outlier, and rotamer regions, respectively (Figure 4G).

### 3.4 Prediction of conformational B cell epitopes

**Figure 5:**
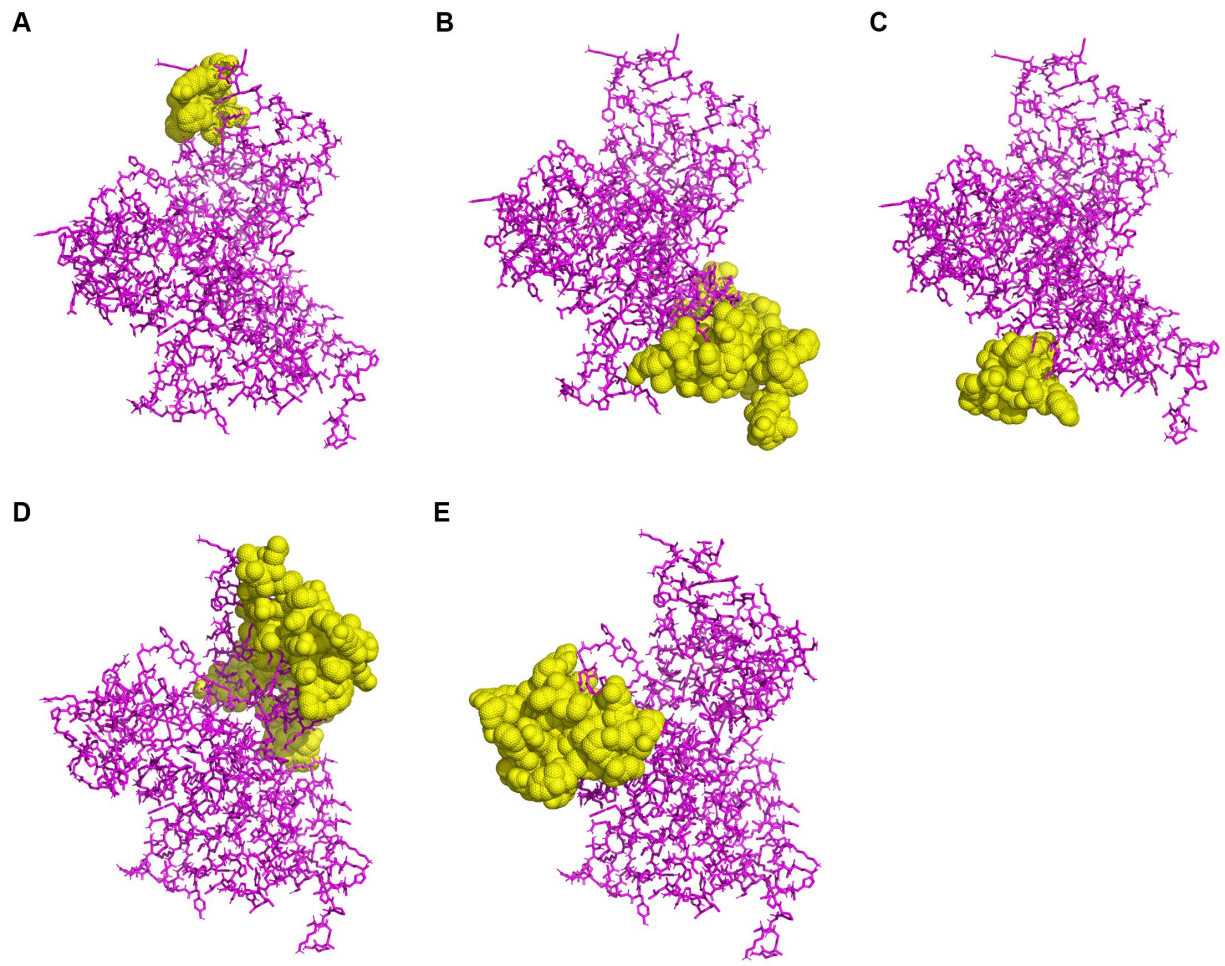
The prediction of conformational B cell epitopes. **(A)** to **(E)** display the five CBEs of *rPPMEV*. The “yellow” regions are the predicted conformational B cell epitopes of *rPPMEV*.

To predict CBEs, the *rPPMEV* was analyzed through the ElliPro server. The results show that there are 238 residues, with values ranging from 0.676 to 0.842, distributed across the eight B cell epitopes in *rPPMEV*. The epitopes range from 11 to 77 amino acid residues, as shown in Figure 5 and Supplementary Table S3.

### 3.5 Molecular docking between *rPPMEV* and TLRs

**Figure 6:**
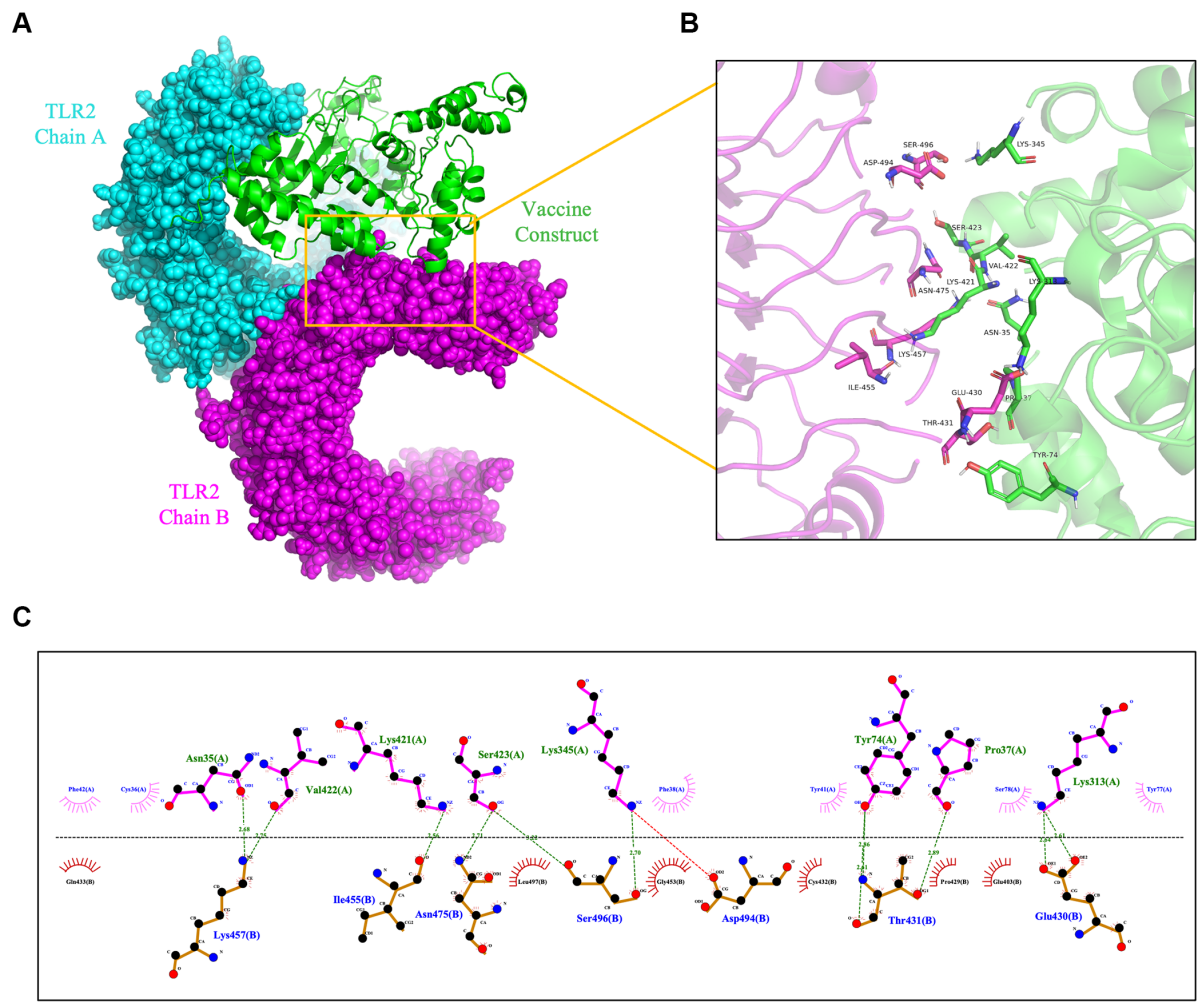
The molecular docking of *rPPMEV* and TLR2. **(A)** The docked complexes of *rPPMEV* and TLR2 with the lowest interaction energy. **(B)** The interaction interface residues of *rPPMEV* and TLR2 predicted by PyMol in 3D. **(C)** The interaction interface residues of *rPPMEV* and TLR2 predicted by Ligplot in 2D. The green dotted line represents the hydrogen bond, and the red dotted line represents the ionic bond.

**Figure 7:**
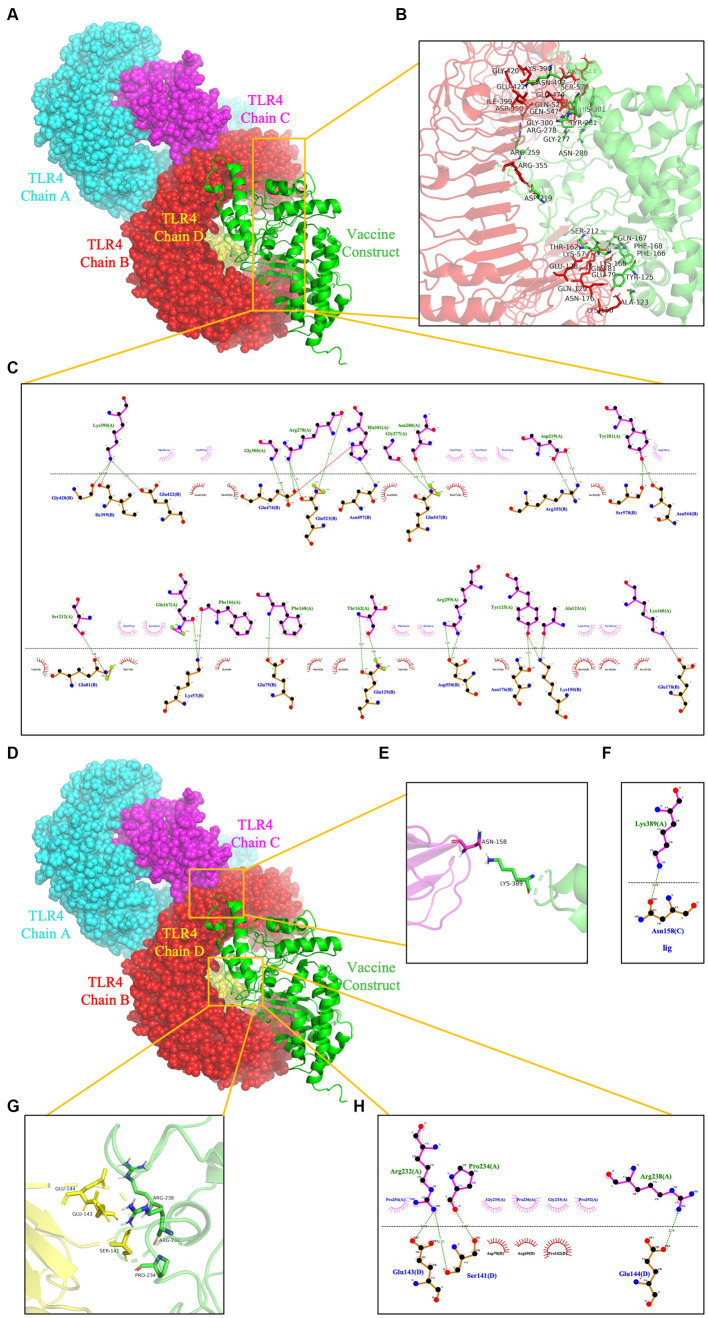
The molecular docking of *rPPMEV* and TLR4. The docked complexes of *rPPMEV* and TLR4 with the lowest interaction energy **(A,D)**. The interaction interface residues predicted by PyMol in 3D of *rPPMEV* with TLR4 Chain B **(B)**, TLR4 Chain C **(E)**, and TLR4 Chain D **(G)**. The interaction interface residues predicted by Ligplot in 2D of *rPPMEV* with TLR4 Chain B **(C)**, TLR4 Chain C **(F)**, and TLR4 Chain D **(H)**. The green dotted line represents the hydrogen bond, and the red dotted line represents the ionic bond.

To prevent and control viruses, the ability of vaccines to induce a brisk and consistent immune response is critical. To achieve the objective of the proposed work, it is necessary to design a vaccine that can interact with the target immune cell receptors (Choudhury et al., 2022). The TLRs are a class of essential protein molecules involved in innate immunity as well as a link between nonspecific and specific immunity (Takeda and Akira, 2004). TLR2 and TLR4 can recognize viral structural glycoproteins, resulting in the production of inflammatory cytokines (Choudhury et al., 2022). To evaluate the interaction and binding consistency between *rPPMEV* and TLRs, molecular docking was performed with the *rPPMEV* ligand and TLR2 as well as TLR4 receptors, respectively. The results show that there are 30 docking results of *rPPMEV*-TLR2 (Supplementary Table S4) and *rPPMEV*-TLR4 (Supplementary Table S5), respectively. The conformation of the docked *rPPMEV*-TLR2 with the lowest interaction energy (−1119.4 kcal/mol) is shown in Figure 6A. The interaction interface residues of this *rPPMEV*-TLR2 complex were analyzed by PyMol in 3D and Ligplot in 2D, respectively. The findings reveal that the complex subunits interact through one ionic bond and 11 hydrogen bonds, as illustrated in Figures 6B,C. Similar to the *rPPMEV*-TLR2, the *rPPMEV*-TLR4 model was selected according to its lowest energy weighted score (−1032.7 kcal/mol), as shown in Figures 7A,D. The interaction interface residue analysis in 3D and 2D formats reveal that there are 2 ionic bonds and 25 hydrogen bonds at the docking interface of *rPPMEV* and the TLR4 Chain B, as shown in Figures 7B,C, one hydrogen bond at the docking interface of *rPPMEV* and the TLR4 Chain C, as shown in Figures 7E,F, and 4 hydrogen bonds at the docking interface of *rPPMEV* and the TLR4 Chain D, as shown in Figures 7G,H. These results indicate that *rPPMEV* has excellent performance in tightly binding to TLR2 and TLR4 to trigger a strong immune response.

### 3.6 Molecular dynamics simulations between *rPPMEV* and TLRs

**Figure 8:**
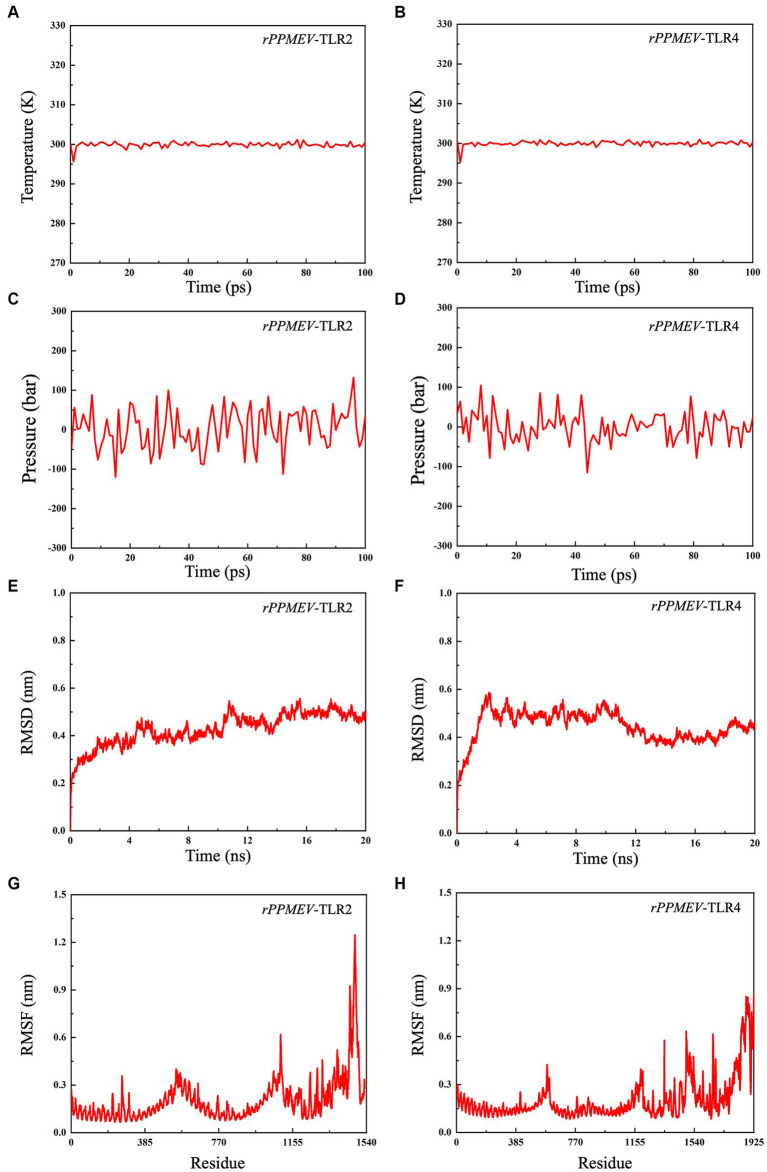
Molecular dynamics simulations between *rPPMEV* and TLRs. The temperature plots of the *rPPMEV*-TLR2 complex **(A)** and the *rPPMEV*-TLR4 complex **(B)**. The pressure plots of the *rPPMEV*-TLR2 complex **(C)** and the *rPPMEV*-TLR4 complex **(D)**. The RMSD analysis of the *rPPMEV*-TLR2 complex **(E)** and the *rPPMEV*-TLR4 complex **(F)**. The RMSF analysis of the *rPPMEV*-TLR2 complex **(G)** and the *rPPMEV*-TLR4 complex **(H)**.

To evaluate the structural stability of the *rPPMEV*-TLR2 and *rPPMEV*-TLR4 complexes, the MD simulation was conducted using GROMACS. The results of MD simulations of the *rPPMEV*-TLR2 and *rPPMEV*-TLR4 complexes are presented in Figure 8. With 100 ps of the time interval, the temperatures of the two simulation systems (*rPPMEV*-TLR2, *rPPMEV*-TLR4) are both around 300 K (Figures 8A,B), and the pressure of the two systems is around 1.4 atmosphere (Figure 8C) and 0.75 atmospheres (Figure 8D), respectively. These results indicate that the system is stable, and the MD operation is successful. In addition, during a 20 ns MD simulation, the RMSD value of the *rPPMEV*-TLR2 complex rises sharply to 0.4 nm in 2 ns and then remains at 0.43 nm (Figure 8E), while the RMSD value of the *rPPMEV*-TLR4 complex reveals a large fluctuation between 0 and 2 ns before being constant around 0.5 nm (Figure 8F). It was reported that the RMSD of the ligand is considered to be fixed within 1 nm, stable below 2 nm, and unstable above 2 nm during molecular docking (Eweas et al., 2021). The RMSD results of *rPPMEV*-TLR2 and *rPPMEV*-TLR4 are both less than 1 nm within 20 ns, indicating that the interaction of the two complexes at the docking interface is fixed. Furthermore, RMSF values demonstrate that the RMSF profiles of most amino acid residues of the *rPPMEV*-TLR2 complex (Figure 8G) and the *rPPMEV*-TLR4 complex (Figure 8H) are below 0.45 nm, and only a few residues have significant changes. These results prove that the two complexes have stability and stiffness.

### 3.7 Immune simulation

**Figure 9:**
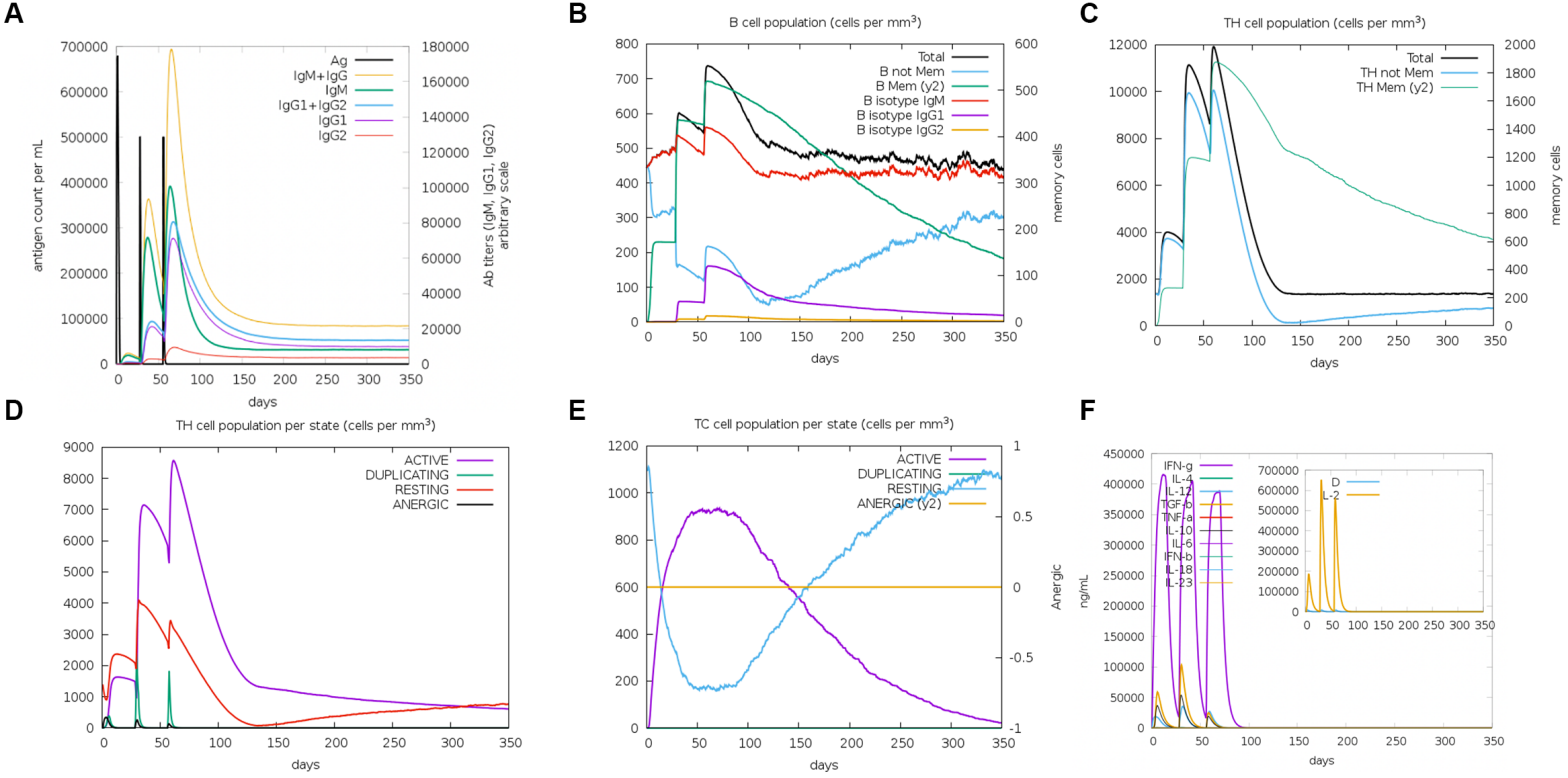
The immune simulated response spectrum of *rPPMEV* in the C-ImmSim server. **(A)** The production of various types of antibodies after vaccination. **(B)** The population of B cells after vaccination. **(C)** The population of helper T cells after vaccination. **(D)** The population of helper T cells in various states. **(E)** The population of cytotoxic T cells in various states. **(F)** Secretion levels of cytokines after vaccination.

As an intracellular pathogen, cellular and humoral immunity induced by vaccines is essential for killing and eliminating viruses. To evaluate the immunological efficacy of *rPPMEV*, the immune stimulation of *rPPMEV* was performed by the C-ImmSim Server. The results show that the *rPPMEV* can induce three peaks in antibody levels after three vaccine doses, as shown in Figure 9A. The antibodies IgM + IgG, IgM, and IgG2 are found in the primary immunization. Further, as immune responses enhance, the levels of total IgM + IgG, IgM, and IgG1 + IgG2 antibodies elevate, indicating that antibody titers increase after the second and third injections. These increasing levels of antibodies in the immune response are mainly attributed to the increase in the total count of B-lymphocytes and T-lymphocytes. As shown in Figure 9B, the B cell population is highly stimulated upon immunization. Apart from B-lymphocytes, *rPPMEV* also induces the formation of three gradually rising peaks in the T helper (TH) (Figure 9C) cell and active TH cell (Figure 9D) populations after three injections, respectively. Moreover, the active cytotoxic T lymphocyte (TC cell) count sustains growth after each immunization (Figure 9E). In the end, as shown in Figure 9F, the concentrations of IFN-γ and IL-2 are both at high levels during each injection, indicating that *rPPMEV* may have the ability to induce a sufficient immune response (Kar et al., 2020).

### 3.8 Codon optimization and *in silico* cloning

**Figure 10:**
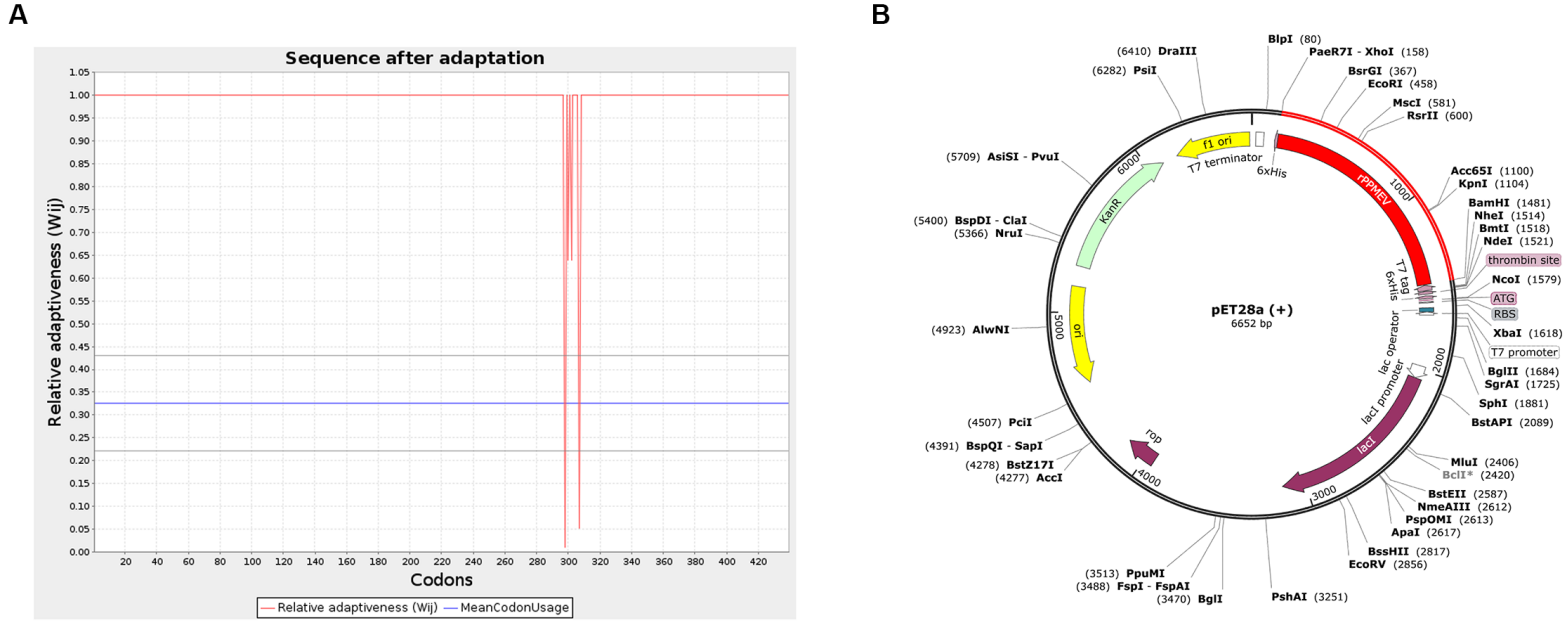
Codon optimization and *in silico* cloning. **(A)** The codon optimization of *rPPMEV*. **(B)** *In silico* cloning of *rPPMEV* in the pET28a (+) vector. The red areas represent the *rPPMEV*, while the black areas represent the pET28a (+) expression vector.

To generate an appropriate plasmid construct harboring the vaccine construct sequence, codon optimization was embarked upon, as shown in Figure 10A. The results show that the improved sequence has a codon adaptation index (CAI) value of 0.98 and a GC content of 50.87, indicating that the protein of the vaccine has a high potential to be well expressed in *E. coli* (Ali et al., 2017). Subsequently, the improved sequence of 1,317 bases was cloned into the pET28a (+) vector between the *XhoI* and *BamHI* restriction sites using Snap-Gene software, as shown in Figure 10B.

## 4 Conclusion

In summary, our study highlights a promising vaccine for PEDV and PDCoV prevention. The vaccine has several advantages. (1) The peptides of the vaccine, derived from the S proteins with the good immune activity of the current common PEDV and PDCoV strains, have a more promising protective effect for the host in either the case of the current epidemic PEDV or PDCoV infection alone or in the case of co-infection than the original peptide molecules for the prevention of PEDV or PDCoV alone. (2) The vaccine, which contains multiple MHC epitopes, the TLR2 agonist Pam2Cys, and The TLR4 agonist RS-09, could target antigen-presenting cells to initiate innate immune responses and provide high levels of either antibody production or cytotoxic cellular response. (3) The vaccine has strong immunogenicity, antigenicity, non-toxicity, and non-sensitization properties. The physicochemical and immunological properties of the vaccine are based on bioinformatics analysis. Although it was reported that the vaccines designed by this method have been proven to produce protective effects *in vivo* and some of them have entered the clinical trial stage (Mahmud et al., 2021), the efficacy evaluation of the vaccine *rPPMEV* still needs to be evaluated by *in vivo* and *in vitro* tests to finally prove the efficacy of this vaccine.

## Data availability statement

The original contributions presented in the study are included in the article/Supplementary material, further inquiries can be directed to the corresponding author.

## Author contributions

WeiH: Conceptualization, Funding acquisition, Investigation, Methodology, Writing – original draft, Writing – review & editing. HeW: Investigation, Writing – review & editing. WW: Investigation, Writing – review & editing. RW: Investigation, Writing – review & editing. WH: Investigation, Writing – review & editing. SW: Investigation, Writing – review & editing. BW: Investigation, Writing – review & editing. HaiW: Conceptualization, Funding acquisition, Investigation, Writing – review & editing.

## Footnotes

^1^https://www.ncbi.nlm.nih.gov/gene/

^2^http://www.cbs.dtu.dk/services/SignalP/

^3^https://www.iedb.org

^4^http://www.ddg-pharmfac.net/vaxijen/VaxiJen/VaxiJen.html

^5^https://services.healthtech.dtu.dk/service.php?NetMHCIIpan-4.0

^6^http://www.ddg-pharmfac.net/AllerTOP

^7^https://web.expasy.org/protparam/

^8^https://npsa-prabi.ibcp.fr/cgi-bin/npsa_automat.pl?page=npsa_sopma.html

^9^https://robetta.bakerlab.org

^10^http://galaxy.seoklab.org/

^11^http://swissmodel/expasy.org/assess

^12^https://prosa.services.came.sbg.ac.at/prosa.php

^13^http://tools.iedb.org/ellipro/

^14^https://cluspro.bu.edu/login.php?redir/queue.php

^15^http://kraken.iac.rm.cnr.it/C-IMMSIM/

^16^https://www.jcat.de/

^17^http://www.snapgene.com/
