# Supplementary material for: Developing a multi-epitope vaccine candidate to combat porcine epidemic diarrhea virus and porcine deltacoronavirus co-infection by employing an immunoinformatics approach
Source: Front Microbiol. 2023 Nov 21;14:1295678. doi: 10.3389/fmicb.2023.1295678 (PMC10703371; doi:10.3389/fmicb.2023.1295678)
Supplement: Supplementary file 1 [file Data_Sheet_1.pdf]

Supplementary Material

1 Supplementary Data

1.1 The amino acid sequence of PEDV S proteins

UUT43943:MKSLTYFWLLLPVLSTLSLPQDVTRCSANTNFRRFFSKFNVQAPAVVVLGGYLPI
GENQGVNSTWYCAGQHPTASGVHGIFLSHIRGGHGFEIGISQEPFDPSGYQLYLHKATNGNT
NATARLRICQFPSIKTLGPTADNDVTTGRNCLFNKAIPAHMSEHSVVGITWDNDRVTVFSDKI
YHFYFKNDWSRVATKCYNSGGCAMQYVYEPTYYMLNVTSAGEDGISYQLCTANCIGYAA
NVFATEPNGHIPEGFSFNNWFLLSNDSTLVHGKVVSNQPLLVNCLLATPKIYGLGQFFSFNQT
IDGVCNGAAVQRAPEALRFNINDTSVILAEGSIVLHTALGTNLSFVCSNSSDPHLATFAIPLGA
TQVPYYCFLKVDTYNSTVYKFLAVLPPNVREIVITKYGDVYVNGFGYLHLGLLDAVTINFTG
HGTDDDVSGFWTIASTNFVDALIEVQGTAIQRILYCDDPVSQLKCSQVSFDLDDGFYPISSRN
LLSHEQPISFVTLPSFNDHSFVNITVSASFGGHSGANLIASDTTINGLSSFCVDTRQFTISLFYN
VTNSYGYVSKSQDSNCPFTLQSVNDYLSFSKFCVSTSLLASACTIDLFGYPEFGSGVKFTSLY
FQFTKGELITGTPKPLEGVTDVSFMTLDVCTKYTIYGFKGEGIITLTNSSILAGVYYTSDSGQL
LAFKNVTSGAVYSVTPCSFSEQAAYVDDDIVGVISSLSSSTFNSTRELPGFFYHSNDGSNCTEP
VLVYSNIGVCKSGSIGYVPSQSGQVKIAPTVTGNISIPTNFSMSIRTEYLQLYNTPVSVDCATY
VCNGNSRCKQLLTQYTAACKTIESALQLSARLESVEVNSMLTISEEALQLATISSFNGDGYNF
TNVLGVSVYDPASGRVVQKRSFIEDLLFNKVVTNGLGTVDEDYKRCSNGRSVADLVCAQY
YSGVMVLPGVVDAEKLHMYSASLIGGMVLGGFTAAAALPFSYAVQARLNYLALQTDVLQR
NQQMLAESFNSAIGNITSAFESVKEAISQTSKGLNTVAHALTKVQEVVNSQGAALTQLTVQL
QHNFQAISSSIDDIYSRLDILSADVQVDRLITGRLSALNAFVAQTLTKYTEVQASRKLAQQKV
NECVKSQSQRYGFCGGDGEHIFSLVQAAPQGLLFLHTVLVPGDFVDVIAIAGLCVNDEIALT
LREPGLVLFTHELQNHTATEYFVSSRRMFEPRKPTVSDFVQIESCVVTYVNLTRDQLPDVIPD
YIDVNKTLDEILASLPNRTGSSLPLDVFNATYLNLTGEIADLEQRSESLRNTTEKLQSLIYNIN
NTLVDLEWLNRVETYIKWPWWVWLIIFIVLIFVVSLLVFCCISTGCCGCCGCCCACFSGCCR
GPRLQPYEVFEKVHVQ

AZL49329:MKSLTYFWLFLPVLSTLSLPQDVTRCSANTNFRRFFSKFNVQAPAVVVLGGYLPI
GENQGVNSTWYCAGQHPTASGVHGIFLSHIRGGHGFEIGISQEPFDPSGYQLYLHKATNGNT
NATARLRICQFPSIKTLGPTADNDVTTGRNCLFNKAIPAHMSEHSVVGITWDNDRVTVFSDKI
YHFYFKNDWSRVATKCYNSGGCAMQYVYEPTYYMLNVTSAGEDGISYQLCTANCIGYAA
NVLATEPNGHIPEGFSFNNWFLLSNDSTLVHGKVVSNQPLLVNCLLAMPKIYGLGQFFSFNQ
TIDGVCNGAAVQRAPEALRFNINDTSVILAEGSIVLHTALGTNLFFVCSNSSDPHLATFTIPLG
ATQVPYYCFLKVDTYNSTVYKFLAVLPPTVREIVITKYGDVYVNGFGYLHLGLLDAVTINFT
GHGTDDDVSGFWTIASTNFVDALIEVQGTAIQRILYCDDPVSQLKCSQVAFDLDDGFYPISSR
NLLSHEQPISFVTPPSFNDHSFVNITVSASFGGHSGANLIASDTTINGFSSFCVDTRQFTISLFYN
VTNSYGYVSKSQDSNCPFTLQSVNDYLSFSKFCVSTSLLASACTIDLFGYPEFGSGVKFTSLY
FQFTKGELITGTPTPLEGVTDVSFMTLDVCTKYTIYGFKGEGIITLTNSSFLAGVYYTSDSGQL
LAFKNVTSGAVYSVTPCSFSEQAAYVDDDIVGVISSLSSSTFNSTRELPGFFYHSNDGSNCTEP
VLVYSNIGVCKSGSIGYVSSQSGQVKIAPTVTGNISIPTNFSMSIRTEYLQLYNTPVSVDCATY
VCNGNSRCKQLLTQYTAACKTIESALQLSARLESVEVNSMLTISEEALQLATISSFNGDGYNF
TNVLGVSVYDPASGRVVQKRSFIEDLLFNKVVTNGLGTVDEDYKRCSNGRSVADLVCAQY

YSGVMVLPGVVDAEKLHMYSASLIGGMVLGGFTSAAALPFSYAVQARLNYLALQTDVLQR
NQQMLAESFNSAIGNITSAFESVKEAISQTSKGLNTVAHALTKVQEVVNSQGAALTQLTVQL
QHNFQAISSSIDDIYSRLDILSADVQVDRLITGRLSALNAFVAQTLTKYTEVQASRKLAQQKV
NECVKSQSQRYGFCGGDGEHIFSLVQAAPQGLLFLHTVLVPGDFVNVIAIAGLCVNDEIALT
LREPGLVLFTHELQDTATEYFVSSRRMYEPRKPTVGDFVQIESCVVTYVNLTRDQLPEVIPDY
IDVNKTLDEILASLPNRTGPSLSLDVFNATYLNLTGEIADLEQRSESLRNTTEELQSLIYNINNT
LVDLEWLNRVETYIKWPWWVWLIIFIVLIFVVSLLVFCCISTGCCGCCGCCGACFSGCCRGP
RLQPYEAFEKVHVQ

UWU45211:MKSLTYFWLLLPVLSTLSLPQDVTRCSANTNFRRFFSKFNVQAPAVVVLGGYLP
IGENQGVNSTWYCAGQHPTASGVHGIFLSHIRGGHGFEIGISQEPFDPSGYQLYLHKATNGNT
NATARLRICQFPSIKTLGPTADNDVTTGRNCLFNKAIPAHMSEHSVVGITWDNDRVTVFSDKI
YHFYFKNDWSRVATKCYNSGGCAMQYVYEPTYYMLNVTSAGEDGISYQLCTANCIGYAA
NVFATEPNGHIPEGFSFNNWFLLSNDSTLVHGKVVSNQPLLVNCLLAMPKIYGLGQFFSFNQ
TIDGVCNGAAVQRAPEALRFNINDTSVILAEGSIVLHTALGTNLSFVCSNSSDPHLATFAIPLG
ATQVPYYCFLKVDTYNSTVYKFLAVLPPNVREIVITKYGDVYVNGFGYLHLGLLDAVTINFT
GHGTDDDVSGFWTIASTNFVDALIEVQGTAIQRILYCDDPVSQLKCSQVSFDLDDGFYPISSR
NLLSHEQPISFVTLPSFNDHSFVNITVSASFGGHSGANLIASDTTINGLSSFCVDTRQFTISLFY
NVTNSYGYVSKSQDSNCPFTLQSVNDYLSFSKFCVSTSLLASACTIDLFGYPEFGSGVKFTSL
YFQFTKGELITGTPKPLEGVTDVSFMTLDVCTKYTIYGFKGEGIITLTNSSILAGVYYTSDSGQ
LLAFKNVTSGAVYSVTPCSFSEQAAYVDDDIVGVISSLSSSTFNSTRELPGFFYHSNDGSNCT
EPVLVYSNIGVCKSGSIGYVPSQSGQVKIAPTVTGNISIPTNFSMSIRTEYLQLYNTPVSVDCA
TYVCNGNSRCKQLLTQYTAACKTIESALQLSARLESVEVNSMLTISEEALQLATISSFNGDGY
NFTNVLGVSVYDPASGRVVQKRSFIEDLLFNKVVTNGLGTVDEDYKRCSNGRSVADLVCAQ
YYSGVMVLPGVVDAEKLHMYSASLIGGMVLGGFTSAAALPFSYAVQARLNYLALQTDVLQ
RNQQMLAESFNSAIGNITSAFESVKEAISQTSKGLNTVAHALTKVQEVVNSQGAALTQLTVQ
LQHNFQAISSSIDDIYSRLDILSADVQVDRLITGRLSALNAFVAQTLTKYTEVQASRKLAQQK
VNECVKSQSQRYGFCGGDGEHIFSLVQAAPQGLLFLHTVLVPGDFVDVIAIAGLCVNDEIAL
TLREPGLVLFTHELQNHTATEYFVSSRRMFEPRKPTVSDFVQIESCVVTYVNLTRDQLPDVIP
DYIDVNKTLDEILASLPNRTGSSLPLDVFNATYLNLTGEIADLEQRSESLRNTTEELQSLIYNI
NNTLVDLEWLNRVETYIKWPWWVWLIIFIVLIFVVSLLVFCCISTGCCGCCGCCCACFSGCC
RGPRLQPYEVFEKVHVQ

1.2 The amino acid sequence of PDCoV S proteins

QZA57171:MQRALLIMTLLCLVRAKFADDLLDLLTFSGAHRFLHKPTSNSSSLYSRANNNFD
VGVLPGYPTKNVNLFSPLTNSTLPINGLHRSYQPLMLNCLTKITNHTLSMYLLPSEIQTYSCG
GAMVKYQTHDAVRIILDLTATDHISLEVVGQHGENYVFVCSEQFNYTTALHNPTFFSLNSEL
YCFTNNTYLGILPPDLTDFTVYRTGQFYANGYLLGTLPITVNYVRLYRGHLSANSAHFALAN
LTDTLITLTNTTISQITYCDKSVVDSIACQRSSHEVEDGFYSDPKSAVRARQRTIVTLPKLPELE
VVHLNISAHMDFGEARLDSVTINGNTSYCVTKPYFRLETNFMCTGCTMNLRTDTCSFDLSA
VNNGMSFSQFCLSTESGACEMKIIVTYVWKYLLRQRLYVTAVEGQTHTGTTSVHATDTSSVI
TDVCTDYTIYGVSGTGIIKPSDLLLHNGIAFTSPTGELYAFKNITTGKTFQVLPCETPSLLIVIN
NTVVGAITSSNSTENNRFTTSIVTPTFFYSTNATTFNCTKPVLSYGPISVCSDGAIVGASTLQNI
RPSIVSLYDGEVEIPSAFSLSVQTEYLQVQADQVIVDCPQYVCNGNSRCLQLLAQYTSACSNI
EAALHSSAQLDSREIISMFKTSTQSLQLANITNFKGDYNFSSLLTTRIGGRSAIEDLLFNKVVT
SGLGTVDQDYKACSRDMAIADLVCSQYYNGIIVLPGVVDAEKMAMYTGSLTGAMVFGGLT
AAAAIPFATAVQARLNYVALQTNVLQENQKILAESFNQAVGNISLALSSVNDAIQQTSEALN

TVAIAIKKIQTVVNQQGEALSHLTAQLSNNFQAISTSIQDIYNRLEEVEANQQVDRLITGRLA
ALNAYVTQLLNQMSQIRQSRLLAQQKINECVKSQSSRYGFCGNGTHIFSLTQTAPNGIFFMH
AVLVPNKFTRVNASAGICVDNTRGYSLQPQLILYQFNNSWRVTPRNMYEPRLPRQADFIQLT
DCSVTFYNTTAANLPNIIPDIIDVNQTVSDIIVNLPTATPPQWDVGIYNNTILNLTVEINDLQER
SKNLSQIADLLQNYIDNLNNTLVNLDWLNRVETYLKWPWYIWLAIALALIAFVTILITIFLCT
GCCGGCFGCCGGCFGLFSKKKRYTDDQPTPSFKFKEW

AXP32216:MQRALLIMTLLCLVRAKFADDLLDLLTFPGAHRFLHKLTSNSSSLYSRANNFDV
GVLPGYPTKNVNLFSPLTNSTLPINGLHRSYQPLMLNCLTKITNHTLSMYLLPSEIQTYSCGG
AMVKYQTHDAVRIILDLTVTDHISVEVVGQHGENYVFVCSEQFNYTTALHNSTFFSLNSELY
CFTNNTYLGILPPDLTDFTVYRTGQFYANGYLLGTLPITVNYVRLYRGHLSANSAHFALANL
TDTLITLTNTTISQITYCDKSVVDSIACQRSSHEVEDGFYSDPKSAVRARQRTIVTLPKLPELE
VVQLNISAHMDFGEARLDSVTINGNTSYCVTKPYFRLETNFMCTGCTMNLRTDTCSFDLSA
VNNGMSFSQFCLSTESGACEMKIIVTYVWNYLLRQRLYVTAVEGQTHTGTTSVHATDTSSVI
TDVCTDYTIYGVSGTGIIKPSDLLLHNGIAFTSPTGELYAFKNITTGKTLQVLPCETPSQLIVIN
NTVVGAITSSNSTENNRFTTTIVTPTFFYSTNATTFNCTKPVLSYGPISVCSDGAIVGTSTLQNT
RPSIVSLYDGEVEIPSAFSLSVQTEYLQVQAEQVIVDCPQYVCNGNSRCLQLLAQYTSACSNI
EAALHSSAQLDSREIINMFQTSTQSLQLANITNFKGDYNFSSILTTRLGGRSAIEDLLFNKVVT
SGLGTVDQDYKACSRDMAIADLVCSQYYNGIMVLPGVVDAEKMAMYTGSLTGAMVFGGL
TAAAAIPFATAVQARLNYVALQTNVLQENQKILAESFNQAVGNISLALSSVNDAIQQTSEAL
NTVAIAIKKIQTVVNQQGEALSHLTAQLSNNFQAISTSIQDIYNRLEEVEANQQVDRLITGRL
AALNAYVTQLLNQMSQIRQSRLLAQQKINECVKSQSSRYGFCGNGTHIFSLTQTAPNGIFFM
HAVLVPNKFTRVNASAGICVDNTRGYSLQPQLILYQFNNSWRVTPRNMYEPRLPRQADFIQL
TDCSVTFYNTTAANLPNIIPDIIDVNQTVSDIIDNLPTATPPQWDVGIYNNTILNLTVEINDLQE
RSKNLSQIADRLQNYIDNLNNTLVDLEWLNRVETYLKWPWYIWLAIALALIAFVTILITIFLC
TGCCGGCFGCCGGCFGLFSKKKRYTDDQPTPSFKFKEW

QCO76963:MQRALLIMTLLCLVRAKFADDLLDLLTFPGAHRFLHKLTSNSSSLHSRANNFDV
GVLPGYATKNVNLFSPLTNSTLPINGLHRSYQPLMLNCFTKITNHTLSMYLLPSEVQTYSCGG
AMVKYQTHDAVRIILDLTATDHISVEVVGQHGENYVFVCSEQFNYTTALHNSTFFSLNSELY
CFTNNTYLGILPPDLTDFTVYRTGQFYANGYLLGTLPITVNYVRLYRGQLAANSAHFALANL
TDTLITLTNTTISQITYCDKSVVDSIACQRSSHEVEDGFYSDPKSAVRARQRTIVTLPKLPELE
VVQLNISAHMDFGEARLDSVTINGNTSYCVTKPYFRLETNFMCTGCTMNLRTDTCSFDLSA
VNNGMSFSQFCLSTESGACEMKIIVTYVWNYLLRQRLYVTAVEGQTHTGTTSVHATDTSSVI
TDVCTDYTIYGVSGTGIIKPSDLLLHNGIAFTSPTGELYAFKNITTGKTLQVLPCETPSQLIVIN
NTVVGAITSSNSTENNRFTTTIVTPTFFYSTNATTFNCTKPVLSYGPISVCSDGAIVGTSTLQNT
RPSIVSLYDGEIEIPSAFSLSVQTEYLQVQAEQVIVDCPQYVCNGNSRCLQLLAQYTSACSNIE
AALHSSAQLDSREIINMFQTSTQSLQLANITNFKGDYNFSSILTTRLGGRSAIEDLLFNKVVTS
GLGTVDQDYKACSRDMAIADLVCSQYYNGIMVLPGVVDAEKMAMYTGSLTGAMVFGGLT
AAAAIPFATAVQARLNYVALQTNVLQENQKILAESFNQAVGNISLALSSVNDAIQQTSEALN
TVAIAIKKIQTVVNQQGEALSHLTAQLSNDFQATSTSIQDIYNRLEEVEANQQVDRLITGRLA
ALNAYVTQLLNQMSQIRQSRLLAQQKINECVKSQSSRYGFCGNGTHIFSLTQTAPNGIFFMH
AVLVPNKFTRVNASAGICVDNTRGYSLQPQLILYQFNNSWRVTPRNMYEPRLPRRADFIQLT
DCSVNFYNTTAANLPNIIPDVIDVNQTVSDIIDNLPTATPPQWDVGIYNNTILNLTVEIKDLQE
RSKNLSQIADRLQNYIDNLNNTLVDLEWLNRVETYLKWPWYIWLAIALALIAFVTILITIFLC
TGCCGGCFGCCGGCFGLFSKKKRYTDDQPTPSFKFKEW

QGZ00525:MQRALLIMTLLCLVRAKFADDLLDLLTFPGAHRFLHKLTSNSSSLYSRANNFDV
GVLPGYATKNVNLFSPLTNSTLPINGLHRSYQPLMLNCFTKITNHTLSMYLLPSDVQTYSCG
GAMVKYQTHDAVRIILDLTATDHISVEVVGQHGENYVFVCSEQFNYTAALHNSTFFSLNSEL
YCFTNNTYLGILPPDLTDFTVYRTGQFYANGYLLGTLPITVNYVRLYRGQLAANSAHFALAN
LTDTLITLTNTTISQITYCDKSVVDSIACQRSSHEVEDGFYSDPKSAVRARQRTIVTLPKLPELE
VVQLNISAHMDFGEARLDSVTINGNTSYCVTKPYFRLETNFMCTGCTMNLRTDTCSFDLSA
VNNGMSFSQFCLSTESGACEMKIIVTYVWNYLLRQRLYVTAVEGQTHTGTTSVHATDTSSVI
TDVCTDYTIYGVSGTGIIKPSDLLLHNGIAFTSPTGELYAFKNITTGKTLQVLPCETPSQLIVIN
NTVVGAITSSNSTENNRFTTTIVTPTFFYSTNATTFNCTKPVLSYGPISVCSDGAIVGISTLQNT
RPSIVSLYDGEIEIPSAFSLSVQTEYLQVQAEQVIVDCPQYVCNGNSRCLQLLAQYTSACSNIE
AALHSSAQLDSREIINMFKTSTQSLQLANITNFKGDYNFSSIITPRIGGRSAIEDLLFNKVVTSG
LGTVDQDYKACSRDMAIADLVCSQYYNGIMVLPGVVDAEKMAMYTGSLTGAMVFGGLTA
AAAIPFATAVQARLNYVALQTNVLQENQKILAESFNQAVGNISLALSSVNDAIQQTSEALNT
VAIAIKKIQTVVNQQGEALSHLTAQLSNNFQAISTSIQDIYNRLEEVEANQQVDRLITGRLAA
LNAYVTQLLNQMSQIRQSRLLAQQKINECVKSQSSRYGFCGNGTHIFSLTQTAPNGIFFMHA
VLVPNKFTRVNASAGICVDNTRGYSLQPQLILYQFNNSWRVTPRNMYEPRLPRQADFIQLTD
CSVTFYNTTAANLPNIIPDVIDVNQTVSDIIDNLPTATPPQWDVGIYNNTILNLTVEINDLQER
SKNLSQIADRLQNYIDNLNNTLVDLEWLNRVETYLKWPWYIWLAIALALIAFVTILITIFLCT
GCCGGCFGCCGGCFGLFSKKKRYTDDQPTPSFKFKEW

QZX45753:MQRALLIMTLLCLVRAKFADDLLDLLTFPGAHRFLHKPTSNSSSHYSRANNFDV
GVLPGYPTKNVNLFSPLTNSTLPINGLHRSYQPLMLNCLTKITNHTLSMYLLPSEIQTYSCGG
AMVKHQTHDAVRIILDLTATDHISVEVVGQHGENYVFVCSEQFNYTTALHNSTFFSLNSELY
CFTNNTYLGILPPDLTDFTVYRTGQFYANGYLLGTLPITVNYVRLYRGHLSANSAHFALANL
TDTLITLTNTTISQITYCDKSVVDSIACQRSSHEVEDGFYSDPKSAVRARQRTIVTLPKLPELE
VVQLNISAHMDFGEARLDSVTINGNTSYCVTKPYFRLETNFMCTGCTMNLRTDTCSFDLSA
VNNGMSFSQFCLSTESGACEMKIIVTYVWNYLLRQRLYVTAVEGQTHTGTTSVHATDTSSVI
TDVCTDYTIYGVSGTGIIKPSDLLLHNGIAFTSPTGELYAFKNITTGKTLQVLPCETPSQLIVIN
NTVVGAITSSNSTENNRFTTTIVTPTFFYSTNATTFNCTKPVLSYGPISVCSDGAIAGTSTLQNT
RPSIVSLYDGEVEIPSAFSLSVQTEYLQVQAEQVIVDCPQYVCNGNSRCLQLLAQYTSACSNI
EAALHSSAQLDSREIINMFQTSTQSLQLANITNFKGDYNFSSILTTRLGGRSAIEDLLFNKVVT
SGLGTVDQDYKACSRDMAIADLVCSQYYNGIMVLPGVVDAEKMAMYTGSLTGAMVFGGL
TAAAAIPFATAVQARLNYVALQTNVLQENQKILAESFNQAVGNISLALSSVNDAIQQTSEAL
NTVAIAIKKIQTVVNQQGEALSHLTAQLSNNFQAISTSIQDIYNRLEEVEANQQVDRLITGRL
AALNAYVTQLLNQMSQIRQSRLLAQQKINECVKSQSSRYCFCGNGTHIFSLTQTAPNGIFFM
HAVLVPNKFTRVNASAGICVDNTRGYSLQPQLILYQFNNSWRVTPRNMYEPRLPRQADFIQL
TDCSVTFYNTTAANLPNIIPDVIDVNQTVSDIIDNLPTATPPQWDVGIYNNTILNLTVEINDLQ
ERSKNLSQIADRLQNYIDNLNNTLVDLEWLNRVETYLKWPWYIWLAIALALIAFVTILITIFL
CTGCCGGCFGCCGGCFGLFSKKKRYTDDQPTPSFKFKEW

2 Supplementary Figures and Tables

2.1 Supplementary Figures

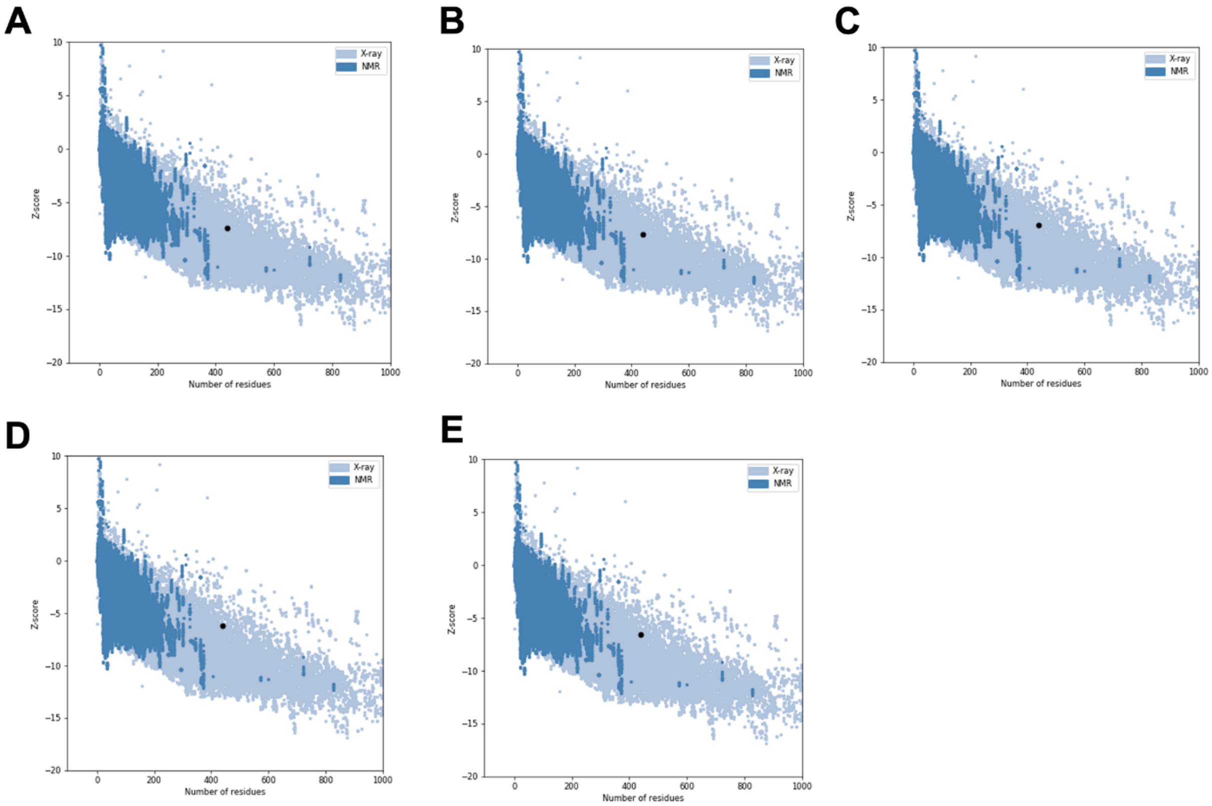

Figure S1. The Z-Score of five models predicted by Robetta software. The Z-Score of Model 1 (A) is -7.43, Model 2 (B) is -7.64, Model 3 (C) is -6.92, Model 4 (D) is -6.19, and Model 5 (E) is -6.59.

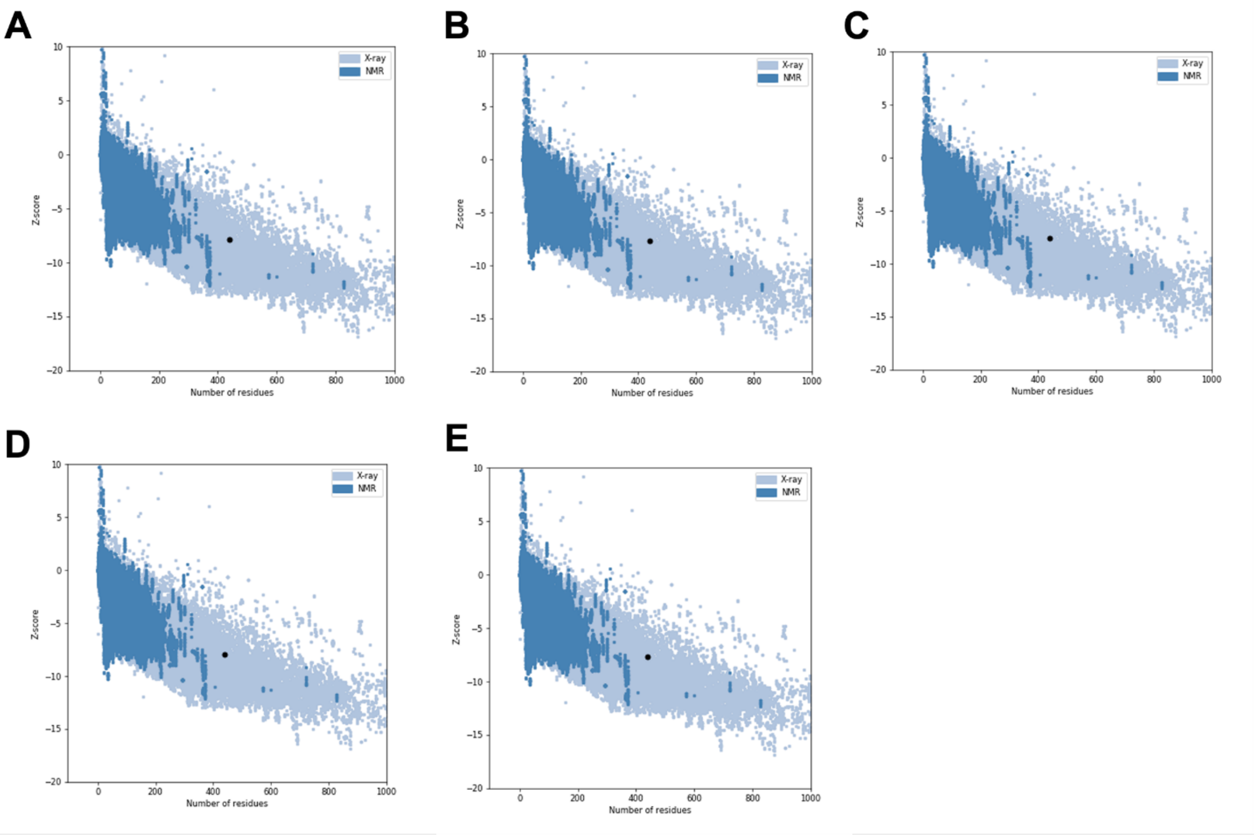

Figure S2. The Z-Score of five optimized models refined by GalaxyRefine Server. The Z-Score of Model 1 (A) is -7.84, Model 2 (B) is -7.69, Model 3 (C) is -7.62, Model 4 (D) is -7.93, and Model 5 (E) is -7.66.

2.2 Supplementary Tables

Table S1. The information on the S proteins of all PEDV and PDCoV strains

	Virus Strain	Protein	Accession Number [a]	Residue Length	Signal Peptide	VaxiJen Score [b]
PEDV	CH/HLJJS/2022	S	UUT43943	1386 aa	1-18 aa	0.4353
	CH/HBXT/2018	S	AZL49329	1385 aa	1-18 aa	0.4283
	SXSL	S	UWU45211	1386 aa	1-18 aa	0.4355
PDCoV	CH-HLJ-20	S	QZA57171	1160 aa	1-19 aa	0.4957
	HNZK-02	S	AXP32216	1159 aa	1-19 aa	0.5022
	Swine/CHN/SC/2018/1	S	QCO76963	1159 aa	1-19 aa	0.5107
	CHN/Sichuan/2019	S	QGZ00525	1159 aa	1-19 aa	0.5014
	CHN-TS1-2019	S	QZX45753	1159 aa	1-19 aa	0.4997

[a] *The accession number from National Center for Biotechnology Information (NCBI).*

[b] *The Virus was selected as the protective antigen prediction model in VaxiJen 2.0, and the default threshold of protective antigen prediction is 0.4.*

Table S2. The antigen epitopes for vaccine construction

Virus	Type	Peptide Sequence	Length	VaxiJen Score[a]
PEDV	CTLs	LLDAVTINF	9	0.9500
		KSQDSNCPF	9	1.1516
		FSMSIRTEY	9	1.5744
		VADLVCAQY	9	1.2280
		YAVQARLNY	9	1.0482
	HTLs	SGVKFTSLYFQFTKG	15	0.9822
		GVKFTSLYFQFTKGE	15	1.2002
		VKFTSLYFQFTKGEL	15	1.2331
		SGQVKIAPTVTGNIS	15	1.0804
	LBEs	RGGHGF	6	0.9275
		INDTSV	6	1.1931
		NSYGYVSKSQDSNCPFTLQSVN	22	0.9706
		YPEFGSG	7	0.9105
		TNFSMSI	7	1.4345
PDCoV	CTLs	SLNSELYCF	9	1.0108
		FSLSVQTEY	9	1.3158
		IADLVCSQY	9	0.9324
		SLQPQLILY	9	0.8543

	GTLPITVNY	9	1.0109
HTLs	EDGFYSDPKSAVRAR	15	0.6250
	RPSIVSLYDGEVEIP	15	0.5657
	ARLNYVALQTNVLQE	15	0.5246
	RLNYVALQTNVLQEN	15	0.5387
LBEs	TFFSLN	6	1.4277
	RPSIVSLY	8	1.2300
	QLDSRE	6	1.5146
	CFGLFSKKKRYTDDQPTPSFK	21	1.1986

[a]*VaxiJen v2.0 was used for predicting antigenicity scores.*

Table S3. The conformational B cell epitopes in the vaccine construct

No.	Residues	Number of Residues	VaxiJen Score[a]
1	A:K381, A:C382, A:F383, A:G384, A:L385, A:F386, A:S387, A:K388, A:K390, A:R391, A:Y392	11	0.842
2	A:E1, A:A2, A:A3, A:A4, A:K5, A:A6, A:P7, A:P8, A:H9, A:A10, A:L11, A:S12, A:E13, A:A14, A:A15, A:A16, A:K17, A:L18, A:L19, A:D20, A:A21, A:I24, A:N25, A:A28, A:Y29, A:S31, A:Q32, A:F42, A:S45, A:I46, A:R47, A:T48, A:E49, A:Y50, A:A51, A:A52, A:Y53, A:V54, A:A55, A:D56, A:L57, A:V58, A:C59, A:A60, A:D104, A:L105, A:V106, A:C107, A:S108, A:Q109, A:Y110, A:A111, A:A112, A:Y113, A:S114, A:L115, A:Q116, A:L119, A:Y134	59	0.732
3	A:A135, A:A136, A:Y137, A:S138, A:G139, A:V140, A:K141, A:F168, A:T169, A:K170, A:G171, A:E172, A:G173, A:P174, A:G175, A:P176, A:G177, A:V178, A:K179, A:F180	20	0.714
4	A:D33, A:S34, A:N35, A:C36, A:N314, A:S315, A:F329, A:T330, A:Q332, A:S333, A:N335, A:K336, A:K337, A:Y338, A:P339, A:E340, A:F341, A:G342, A:S343, A:G344, A:K345, A:K346, A:T347, A:F349, A:R364, A:P365, A:S366, A:S369, A:L370, A:K373, A:Q374, A:D376, A:S377, A:R378, A:K380, A:T393, A:D394, A:D395, A:Q396, A:P397, A:T398, A:P399, A:S400, A:F401, A:K402, A:E403, A:A404, A:A405, A:A406, A:K407, A:F408, A:N409, A:N410, A:F411, A:T412, A:V413, A:V419, A:P420, A:K421, A:V422, A:S423, A:A424, A:S425, A:H426, A:L427, A:E428, A:T429, A:G430, A:A431, A:L432, A:L433, A:A434, A:A435, A:G436, A:A437, A:A438, A:A439	77	0.696
5	A:P194, A:G195, A:P196, A:G197, A:S198, A:G213, A:P214, A:G215, A:P216, A:G217, A:E218, A:D219, A:G220, A:F221, A:Y222, A:S223, A:D224, A:P225, A:K226, A:S227, A:A228, A:V229, A:A231, A:R232, A:G233, A:P234, A:G235, A:P236, A:G237, A:R238, A:P239, A:S240, A:I241, A:V242, A:S243, A:Y245, A:D246, A:G247, A:E248, A:V249, A:E250, A:I251, A:P252, A:G253, A:P254, A:G255, A:P256, A:G257, A:A258, A:R259, A:L260, A:N261, A:Y262, A:V263, A:N268, A:V269, A:L270, A:Q271, A:E272, A:G273, A:P274, A:G275, A:P276, A:G277, A:R278, A:N280, A:Y281, A:V282, A:A283, A:L284, A:Q285	71	0.676

[a] *VaxiJen v2.0 was used for predicting antigenicity scores.*

Table S4. The result of molecular docking between *rPPMEV* and TLR2

Cluster	Members	Representative	Weighted Score
0	49	Center	-1017.8
		Lowest Energy	-1050.6
1	33	Center	-904.2
		Lowest Energy	-1119.4
2	29	Center	-860.4
		Lowest Energy	-972.2
3	29	Center	-752.8
		Lowest Energy	-816.0
4	26	Center	-691.5
		Lowest Energy	-813.8
5	20	Center	-733.7
		Lowest Energy	-838.7
6	20	Center	-923.7
		Lowest Energy	-923.7
7	20	Center	-704.0
		Lowest Energy	-768.3
8	20	Center	-693.9
		Lowest Energy	-854.2
9	17	Center	-727.4
		Lowest Energy	-817.7
10	17	Center	-806.7
		Lowest Energy	-806.7
11	16	Center	-876.9
		Lowest Energy	-876.9
12	16	Center	-719.2
		Lowest Energy	-732.6
13	15	Center	-826.0
		Lowest Energy	-826.0
14	15	Center	-766.1
		Lowest Energy	-807.7
15	14	Center	-716.4
		Lowest Energy	-783.9
16	13	Center	-723.6
		Lowest Energy	-723.6
17	12	Center	-784.2
		Lowest Energy	-784.2
18	12	Center	-757.7
		Lowest Energy	-757.7
19	12	Center	-753.5
		Lowest Energy	-776.2
20	12	Center	-732.5
		Lowest Energy	-732.5
21	11	Center	-695.7
		Lowest Energy	-769.9

22	11	Center	-853.8
		Lowest Energy	-888.3
23	11	Center	-771.2
		Lowest Energy	-787.1
24	10	Center	-734.4
		Lowest Energy	-749.1
25	10	Center	-700.2
		Lowest Energy	-776.1
26	10	Center	-729.1
		Lowest Energy	-729.1
27	10	Center	-726.6
		Lowest Energy	-726.6
28	10	Center	-704.3
		Lowest Energy	-729.4
29	7	Center	-737.9
		Lowest Energy	-850.9

Table S5. The result of molecular docking between *rPPMEV* and TLR4

Cluster	Members	Representative	Weighted Score
0	60	Center	-834.2
		Lowest Energy	-1012.2
1	58	Center	-858.3
		Lowest Energy	-1008.1
2	36	Center	-1007.8
		Lowest Energy	-1007.8
3	35	Center	-817.4
		Lowest Energy	-971.3
4	31	Center	-828.2
		Lowest Energy	-901.9
5	25	Center	-759.8
		Lowest Energy	-880.3
6	21	Center	-907.3
		Lowest Energy	-907.3
7	21	Center	-829.1
		Lowest Energy	-829.1
8	19	Center	-840.4
		Lowest Energy	-853.8
9	19	Center	-907.1
		Lowest Energy	-1032.7
10	19	Center	-883.4
		Lowest Energy	-923.1
11	19	Center	-811.2
		Lowest Energy	-811.2
12	18	Center	-770.3
		Lowest Energy	-908.8
13	18	Center	-785.7
		Lowest Energy	-869.8
14	18	Center	-776.9
		Lowest Energy	-858.5
15	17	Center	-811.6
		Lowest Energy	-940.6
16	17	Center	-854.2
		Lowest Energy	-854.2
17	17	Center	-843.4
		Lowest Energy	-889.9
18	17	Center	-787.2
		Lowest Energy	-859.8
19	16	Center	-755.0
		Lowest Energy	-775.5
20	16	Center	-851.0
		Lowest Energy	-851.0
21	15	Center	-780.7
		Lowest Energy	-835.9

22	15	Center	-760.4
		Lowest Energy	-863.2
23	13	Center	-789.9
		Lowest Energy	-897.5
24	13	Center	-876.8
		Lowest Energy	-876.8
25	13	Center	-861.0
		Lowest Energy	-861.0
26	12	Center	-855.2
		Lowest Energy	-855.2
27	12	Center	-804.6
		Lowest Energy	-804.6
28	10	Center	-842.5
		Lowest Energy	-865.2
29	10	Center	-811.9
		Lowest Energy	-811.9
